# Efficacy and safety of FLT3 inhibitors in monotherapy of hematological and solid malignancies: a systemic analysis of clinical trials

**DOI:** 10.3389/fphar.2024.1294668

**Published:** 2024-05-17

**Authors:** Yuying Zhao, Xuedi Zhang, Xiaoyan Ding, Ying Wang, Zhenpeng Li, Ronglan Zhao, Hai-En Cheng, Yanli Sun

**Affiliations:** School of Medical Laboratory, Shandong Second Medical University, Weifang, China

**Keywords:** FLT3 inhibitor, hematological malignancies, solid tumors, safety, efficacy

## Abstract

**Introduction:** FLT3 mutations are closely associated with the occurrence of hematological and solid malignancies, especially with acute myeloid leukemia. Currently, several FLT3 inhibitors are in clinical trials, and some have been applied in clinic. However, the safety, efficacy and pharmacodynamics of these FLT3 inhibitors have not been systemically analyzed before.

**Methods: **We searched and reviewed clinical trial reports on the monotherapy of 13 FLT3 inhibitors, including sorafenib, lestaurtinib, midostaurin, gilteritinib, quizartinib, sunitinib, crenolanib, tandutinib, cabozantinib, pexidartinib, pacritinib, famitinib, and TAK-659 in patients with hematological and solid malignancies before May 31, 2023.

**Results: **Our results showed the most common adverse events (AEs) were gastrointestinal adverse reactions, including diarrhea, hand-foot syndrome and nausea, while the most common hematological AEs were febrile neutropenia, anemia, and thrombocytopenia. Based on the published data, the mean overall survival (OS) and the mean progression-free survival (PFS) were 9.639 and 5.905 months, respectively. The incidence of overall response rate (ORR), complete remission (CR), partial response (PR), and stable disease (SD) for all these FLT3 inhibitors was 29.0%, 8.7%, 16.0%, and 42.3%, respectively. The ORRs of FLT3 inhibitors in hematologic malignancies and solid tumors were 40.8% and 18.8%, respectively, indicating FLT3 inhibitors were more effective for hematologic malignancies than for solid tumors. In addition, time to maximum plasma concentration (*T_max_*) in these FLT3 inhibitors ranged from 0.7-12.0 hours, but the elimination half-life (*T_1/2_*) range was highly variable, from 6.8 to 151.8 h.

**Discussion: **FLT3 inhibitors monotherapy has shown significant anti-tumor effect in clinic, and the effectiveness may be further improved through combination medication.

## Introduction

The mechanisms underlying the development of cancers are closely associated with gene mutations, which lead to the excessive proliferation and/or impaired differentiation of blast cells (Zou et al., 2022). FLT3 (FMS-like receptor tyrosine kinase 3), a member of the type III receptor tyrosine kinase family, represents one of the most frequently identified mutated genes that disturb cell proliferation and differentiation through interfering intracellular signaling networks in hematologic and solid malignancies (Sun et al., 2020; Zhao et al., 2022).

FLT3, located on 13q12, encodes a 933 amino acid transmembrane receptor, whose molecular weight is 155–160 kDa. It mainly comprises five domains: an ectodomain consisting of five immunoglobulin-like (Ig-like) domains denominated D1, D2, D3, D4, and D5: D1, D2 and D3 are required for the binding of FLT3 ligand (FLT3LG), and D4 and D5 for receptor dimerization (Kazi and Rönnstrand, 2019). These domains are required for cell surface recognition, FLT3/FLT3LG interaction and consequent receptor dimerization. The functional domains comprise: a transmembrane domain with unknown function; a juxtamembrane domain (JMD) regulating the activity of tyrosine kinase, is composed of a binding motif (Y572 to M578), a switch motif (V579 to V592) and linker peptide (D593 to W603); two tyrosine kinase domains (TKDs), TKD1 and TKD2, are separated by a kinase insert region and controlled by the activation loop (Grafone et al., 2012). The mutational hotspots of FLT3 are mainly located in the juxtamembrane region and the activation loop (Takahashi, 2011).

FLT3 mutations occur in approximately 30% of newly diagnosed acute myeloid leukemia (AML) cases (Zhao et al., 2022). The most common type of FLT3 mutations is internal tandem repeats (ITD) in the JMD and point mutations in the TKDs (Takahashi, 2011). Previous studies suggested that FLT3 was the only type III tyrosine kinase that develops ITD (Takahashi, 2011). These mutations lead to the constitutive receptor activation and constant activation of the downstream signaling cascades. Generally, the binding of FLT3LG to the mutated FLT3 leads to excessive activation of PI3K (phosphoinositide 3 kinase) and MAPK (mitogen-activated protein kinase) signaling pathways (Takahashi, 2011; Zhao et al., 2022). In AML, overactivated PI3K phosphorylates AKT1, then the latter promotes the formation of the MDM2-TP53 complex (Zou et al., 2022), the phosphorylation of BCL2 (B-Cell CLL/Lymphoma 2) and BAD (Bcl2 Antagonist Of Cell Death), and the expression of MCL1 (Myeloid Cell Leukemia Sequence 1), which together cause uncontrolled proliferation and decreased differentiation of immature myeloid blast cells (Zhao et al., 2022), while MAPK is involved in the development of AML through enhancing the phosphorylation of ERK1/2 (Takahashi, 2011). Except that, FLT3-ITD still directly activates STAT5, which is independent of JAK or Src kinases (Lv et al., 2021). Therefore, the therapy targeting FLT3 proteins is a promising strategy for certain types of cancers.

Since the first FLT3 inhibitors sorafenib and sunitinib were approved by U.S. Food and Drug Administration (FDA) in 2005 (Escudier et al., 2007) and 2006 (Goodman et al., 2007), respectively, a variety of new FLT3 inhibitors have been developed. Tyrosine kinase inhibitors (TKIs) are small molecules which compete with the ATP binding site of catalytic domain of several oncogenic tyrosine kinases (Yazdi et al., 2017). According to the mode of binding to FLT3, the FLT3 inhibitors are grouped into two types: Type I and II inhibitors (Senapati and Kadia, 2022). The Type I inhibitors bind to the gatekeeper domain close to the activation loop or the ATP-binding pocket of FLT3, which are not affected by its conformation, while Type II inhibitors bind adjacent to the ATP binding domain in the hydrophobic region when the protein is in an inactive conformational state (Senapati and Kadia, 2022). Additionally, type II inhibitors have a better inhibitory effect on FLT3-ITD mutations than FLT3-TKD mutations (Senapati and Kadia, 2022). A diverse range of efficacy and side effects from FLT3 inhibitors has been reported in different studies. In this study, we analyzed the published clinical trials and summarized the safety, efficacy and pharmacokinetics of FLT3 inhibitors including sorafenib, lestaurtinib, midostaurin, gilteritinib, quizartinib, sunitinib, crenolanib, tandutinib, cabozantinib, pexidartinib, pacritinib, TAK-659 (mivavotinib) and famitinib (Supplementary Figure S1).

## Methods

### Study design, search strategy, and study selection

Our study was guided by the Preferred Reporting Items for Systematic Evaluation and Meta-Analysis (PRISMA) (Kolaski et al., 2023), a statement as a guide, and was registered at PROSPERO (CRD42022332826). The problem population, interventions, comparison, and outcomes (PICO) format rules mentioned here were organized: 1) patients with malignancies; 2) interventions: treatment with one of the FLT3 inhibitors; 3) comparison: with or without control; 4) outcomes: adverse events (AEs), efficacy including event-free survival (EFS), progression-free survival (PFS), overall survival (OS), duration Of Therapy (DOT), partial response (PR), complete remission (CR), stable disease (SD), overall response rate (ORR), progressive disease (PD) and pharmacodynamics after drug use, including *T*_max_ (time to maximum plasma concentration) and *T*_*1/2*_ (elimination half-life). The literature search was performed in PubMed, Embase and Cochrane Library databases (by 31 May 2023). Search keywords were FLT3 inhibitors, sorafenib, lestaurtinib, midostaurin, gilteritinib, quizartinib, sunitinib, crenolanib, tandutinib, cabozantinib, pexidartinib, pacritinib, famitinib, TAK-659, and derived combinations without any filters.

The quality of the included studies was assessed using the Methodological Index (MINORS) (Zeng et al., 2015) and the Cochrane risk of bias tool for non-randomized and randomized trials, respectively.

### Inclusion and exclusion criteria

The eligibility criteria in the studies were as follows: 1) clinical trials; 2) patients with malignancies enrolled in these trials were identified through the appropriate diagnostic criteria; 3) the patients were treated with one of FLT3 inhibitors alone regardless of any prior treatment; 4) complete data on safety and/or efficacy were provided in the article. The exclusion criteria were as follows: 1) cellular experiments or animal experiments; 2) articles without original data; 3) articles sharing the same original data; 4) treatment with FLT3 inhibitor and the other drugs simultaneously.

### Data extraction

The extracted data were as follows: 1) basic information including the name of FLT3 inhibitor, the first author, registration number and phase of the clinical trial, publication date, the number, age, and cancer type; 2) characteristics of adverse events (AEs); 3) survival indicators including PFS, EFS, OS, ORR, CR, PR and SD; 4) pharmacodynamics including *T*_max_ and *T*_*1/2*_.

### Analysis of target genes

The target genes of these FLT3 inhibitors in the published articles were collected and summarized.

### Statistical analysis

We analyzed data on survival and AEs using the Comprehensive Meta-Analysis program (CMA 3.0). Event rates and 95% confidence intervals (CI) for survival and AEs were assessed using a statistical threshold of *p* < 0.05. In the statistical analysis, a random-effects model was used if *I* ^*2*^ ≥ 50% and *p* < 0.05, and otherwise a fixed-effects model was applied.

## Results

### Literature search

**FIGURE 1:**
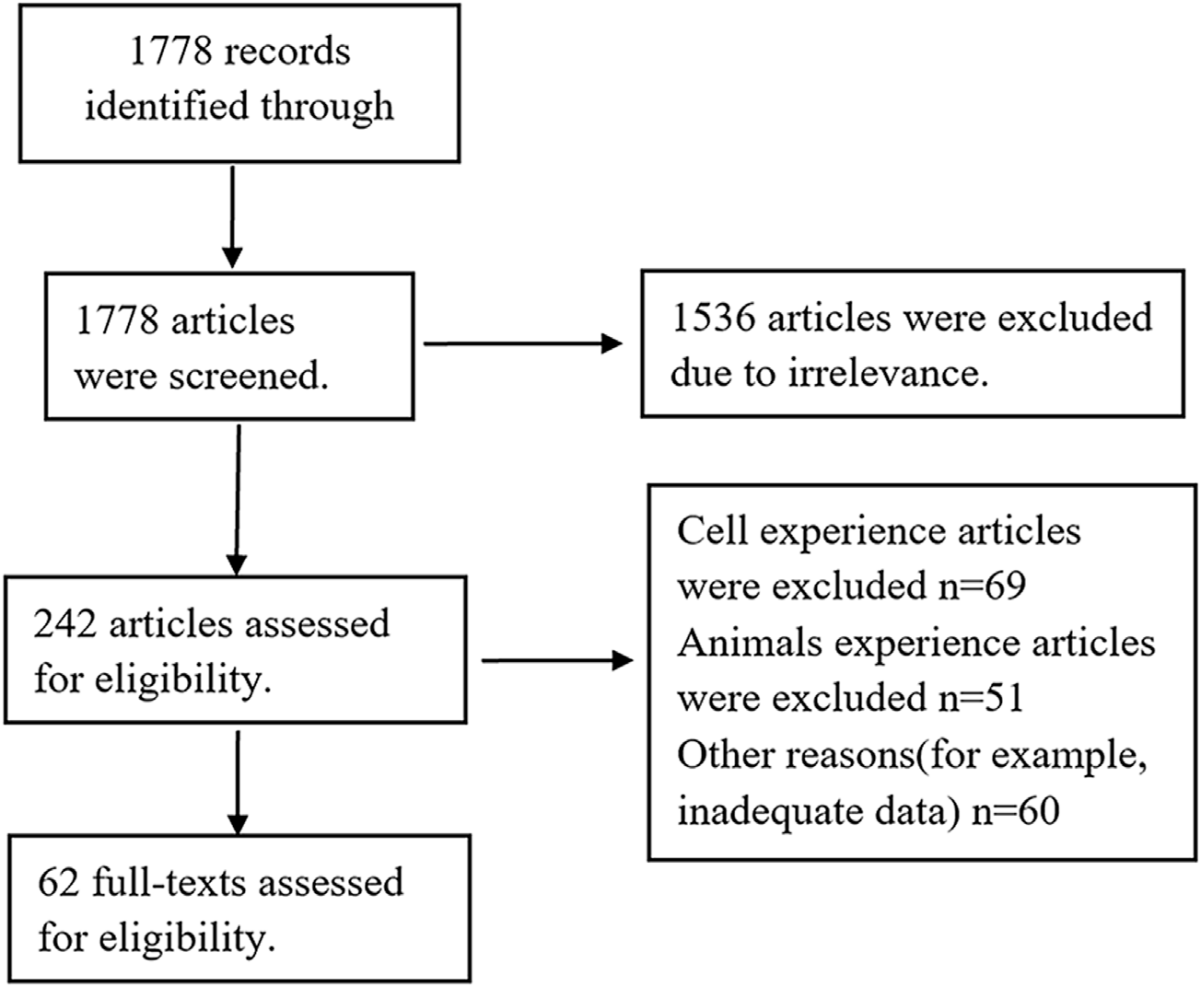
Flow chart of the literature search and selection process.

By 31 May 2023, 1778 potentially relevant articles were obtained by searching PubMed, Embase, and Cochrane Library databases. There were 1,536 articles that were excluded after an initial reading of the articles due to the irrelevance. After careful evaluation of the remaining articles, an additional 242 articles were rejected for non-clinical trials or other reasons. Finally, 62 articles with 4,600 patients totally were included in this study. The screening protocols are shown in Figure 1, while the basic information of the selected studies is shown in Table 1. The youngest and oldest patients were 4 and 97 years old, respectively. Till now, sorafenib, sunitinib, cabozantinib, midostaurin, gilteritinib and pexidartinib have been approved by FDA of USA on 20 December 2005, 26 January 2006, 29 November 2012, 28 April 2017, 28 November 2018, and 2 August 2019, respectively. Moreover, the other FLT3 inhibitors, for example, quizartinib, pacritinib have been in phase Ⅲ. Here, 51 single-arm studies and 11 double-arm studies were included. Expect that crenolanib with insufficient data, the AEs on the other 12 FLT3 inhibitors were evaluated here.

**TABLE 1:** Basic information of the selected articles.

FLT inhibitor	Author	Clinical trial registration number	Phase	Publication year	Number of patients	Study design	Cancer type	Median age (range)
sorafenib	Borthakur G (Borthakur et al., 2011)	NCT00217646	Ⅰ	2011	50	single-arm	AML (n = 48), CMML (n = 1), biphenotypic leukemia (n = 1)	60 (21–88)
sorafenib	Burchert A (Burchert et al., 2020)	DRKS00000591	Ⅱ	2020	43	double-arm	FLT3-ITD–positive AML (n = 43)	54.17 (23.58–74.58)
sorafenib	Chen YB (Chen et al., 2014)	NCT01398501	Ⅰ	2014	22	single-arm	FLT3-ITD AML (n = 22)	54 (20–67)
sorafenib	Semrad TJ (Semrad et al., 2012)	NCT00810394	Ⅱ	2012	50	single-arm	non-small cell lung cancer (n = 15), colorectal cancer (n = 7), head and neck cancer (n = 4), pancreatic cancer (n = 3), soft tissue sarcoma (n = 3), hepatocellular cancer (n = 2), differentiated thyroid cancer (n = 2), gastric cancer (n = 2), adenoid cystic carcinoma (n = 2), prostate cancer (n = 2), renal cell cancer (n = 1), breast cancer (n = 1), testicular cancer (n = 1), mesothelioma (n = 1), bladder cacner (n = 1), melanoma (n = 1), thymic carcinoma (n = 1), ovarian cancer (n = 1)	61 (25–88)
sorafenib	Lin SM (Lin et al., 2017)	NCT01098760	IV	2017	151	single-arm	advanced HCC (n = 151)	62.0 (28–97)
sorafenib	Fierro-Maya LF (Fierro-Maya et al., 2021)	NCT02084732	Ⅱ	2021	19	single-arm	advanced thyroid carcinoma (n = 19)	61.8 (38–84)
sorafenib	Huh KY (Huh et al., 2021)	—	No mention	2021	30	single-arm	healthy male subjects (n = 30)	30.9
sorafenib	Awada A (Awada et al., 2005)	—	Ⅰ	2005	44	single-arm	colon tumor (n = 15), breast tumor (n = 7), kidney tumor (n = 7), ovary tumor (n = 1), liver tumor (n = 1), gastrointestinal tumor (n = 2), head and neck tumor (n = 1), lung tumor n = 1, melanoma (n = 2), unknown tumor (n = 5), other tumor (n = 2)	58 (42–79)
sorafenib	Li D (Li et al., 2022)	NCT03434379	Ⅲ	2022	156	double-arm	locally advanced metastatic or unresectable HCC (n = 156)	64.4 (33–87)
sorafenib	Kudo M (Kudo et al., 2023)	NCT01761266	Ⅲ	2023	476	double-arm	Unresectable HCC (n = 476)	62.0 (22–88)
lestaurtinib	Knapper S (Knapper et al., 2006)	—	Ⅱ	2006	29	single-arm	older patients with AML (FLT3-ITD mutation n = 2, FLT3-TKD mutation n = 3, FLT3 wild type n = 24)	73 (67–82)
lestaurtinib	Smith BD (Smith et al., 2004)	—	Ⅰ/Ⅱ	2004	17	single-arm	relapsed (n = 7) or refractory (n = 10) AML	61 (18–74)
lestaurtinib	Marshall JL (Marshall et al., 2005)	—	Ⅰ	2005	30	single-arm	prostate cancer (n = 5), colorectal cancer (n = 3), renal cancer (n = 4), pancreas cancer (n = 4), lung cancer (n = 4), other cancers (n = 10)	58.7 (29–81)
midostaurin	Fischer T (Fischer et al., 2010)	NCT00045942	Ⅱ	2010	95	single-arm	AML or MDS with either wild-type (n = 60) or mutated (n = 35) FLT3	—
midostaurin	Propper DJ (Propper et al., 2001)	—	Ⅰ	2001	32	single-arm	colon cancer (n = 11), adenocarcinoma unknown primary cancer (n = 4), breast cancer (n = 3), melanoma (n = 2), other cancers (n = 12)	62 (36–76)
midostaurin	He H (He et al., 2017)	—	No mention	2017	6	single-arm	healthy subjects (n = 6)	22–51
gilteritinib	Perl AE (Perl et al., 2019)	NCT02421939	Ⅲ	2019	247	double-arm	patients with R/R AML (n = 247)	62 (20–84)
gilteritinib	Numan Y (Numan et al., 2022)	—	Ⅱ	2022	113	single-arm	R/R FLT3 mutated AML (n = 113)	58.3 (18–92)
gilteritinib	Usuki K (Usuki et al., 2018)	NCT02181660	Ⅰ	2018	24	single-arm	R/R AML (n = 24)	70.5 (60–81)
gilteritinib	Hosono N (Hosono et al., 2021)	NCT02421939	Ⅲ	2021	33	double-arm	FLT3-mutated R/R AML (n = 33)	60 (22–84)
gilteritinib	Perl AE (Perl et al., 2017)	NCT02014558	Ⅰ/Ⅱ	2017	252	single-arm	AML (n = 252)	59
gilteritinib	Dumas PY (Dumas et al., 2023)	NCT05193448	Ⅲ	2023	140	single-arm	FLT3-ITD and/or TKD mutated AML (n = 140)	65.2 (18.2–84.8)
quizartinib	Cortes J (Cortes et al., 2018a)	NCT00989261	Ⅱ	2018	332	single-arm	primary or secondary AML (n = 332)	63 (19–86)
quizartinib	Cortes JE (Cortes et al., 2018b)	NCT01565668	Ⅱb	2018	76	single-arm	secondary AML (n = 10), primary AML (n = 66)	55 (19–77)
quizartinib	Cortes JE (Cortes et al., 2019)	NCT02039726	Ⅲ	2019	245	double-arm	FLT3-ITD primary AML or AML	55 (46–65)
							secondary to MDS (n = 245)	
quizartinib	Usuki K (Usuki et al., 2019)	NCT02675478	Ⅰ	2019	16	single-arm	R/R AML (n = 16)	68 (33–91)
quizartinib	Li J (Li et al., 2020)	No mention	No mention	2020	64	single-arm	healthy subjects (n = 64)	34 (18–55)
sunitinib	Fiedler W (Fiedler et al., 2005)	—	Ⅰ	2005	15	single-arm	refractory or resistant AML (n = 15)	72 (54–80)
sunitinib	Jo JC (Jo et al., 2014)	—	Ⅱ	2014	19	single-arm	advanced aggressive fibromatosis (n = 19)	30 (22–67)
sunitinib	Balaña C (Balaña et al., 2014)	NCT01100177	Ⅱ	2014	12	single-arm	newly diagnosed, non-resectable glioblastoma (n = 12)	65 (48–70)
sunitinib	AI Baghdadi T (Al et al., 2020)	NCT02693535	Ⅱ	2020	10	single-arm	metastatic colorectal cancer with FLT3 amplification (n = 10)	56 (41–71)
sunitinib	DuBois SG (DuBois et al., 2012)	—	Ⅰ	2012	12	single-arm	high-grade glioma (n = 5), brain stem glioma (n = 4), ependymoma (n = 1), mesothelioma (n = 1), undifferentiated carcinoma (n = 1)	13 (4–21)
sunitinib	Britten CD (Britten et al., 2008)	—	Ⅰ	2008	12	single-arm	colorectal tumor (n = 2), gastrointestinal stromal tumor (n = 2), neuroendocrine tumor (n = 2), thyroid tumor (n = 2), angiosarcoma tumor (n = 1), larynx tumor (n = 1), hepatocellular tumor (n = 1), pancreas tumor (n = 1)	57 (28–75)
sunitinib	O'Farrell AM (O'Farrell et al., 2003)	—	Ⅰ	2003	29	single-arm	AML (n = 29)	67 (19–82)
sunitinib	Faivre S (Faivre et al., 2006)	—	—	2006	28	single-arm	renal cell carcinoma (n = 4), neuroendocrine tumors (n = 4), colorectal cancer (n = 3), non–small-cell lung cancer (n = 2), mesotheliomas (n = 2), uterine carcinoma (n = 2), breast cancer (n = 2), pancreas adenocarcinoma (n = 2), angiosarcoma (n = 2), esophagus carcinoma (n = 1), undifferentiated carcinoma of nasopharynx (n = 1), parotid adenocarcinoma (n = 1), melanoma (n = 1), gastrointestinal stromal tumor (n = 1)	55 (33–78)
crenolanib	Cortes JE (Cortes et al., 2016)	NCT01657682/NCT01522469	Ⅱ	2016	69	single-arm	refractory/relapsed FLT3^+^ AML (ITD n = 29, D835 n = 11, ITD + D835 n = 29)	
crenolanib	Collins R (Collins et al., 2014)	NCT01522469/NCT01657682	Ⅱ	2014	19	single-arm	R/R FLT3 mutant AML (n = 19)	47 (21–81)
crenolanib	Lewis NL (Lewis et al., 2009)	—	Ⅰ	2009	59	single-arm	colon cancer (n = 10), connective/soft tissue tumor (n = 7), bronchus/lung tumor (n = 5), ovary tumor (n = 5), other tumor (n = 32)	58.6 (18–80)
tandutinib	Grossman SA (NCT00379080, 2017)	NCT00379080	Ⅰ/Ⅱ	2017	56	single-arm	recurrent or progressive glioblastoma (n = 56)	56 (24–77)
tandutinib	Shepard DR (Shepard et al., 2012)	—	II	2012	10	single-arm	mRCC refractory to previous therapy with sunitinib or sorafenib (n = 10)	61 (55–78)
tandutinib	DeAngelo DJ (DeAngelo et al., 2006)	—	I	2006	40	single-arm	AML (n = 39), high-risk MDS (n = 1)	70.5 (22–90)
cabozantinib	Fathi AT (Fathi et al., 2018)	NCT01961765	Ⅰ	2018	18	single-arm	R/R AML (n = 18)	68 (27–85)
cabozantinib	Matulonis UA (Matulonis et al., 2019)	NCT01716715	Ⅱ	2019	57	double-arm	persistent or recurrent epithelial ovarian, fallopian tube or primary peritoneal cancer (n = 57)	—
cabozantinib	Nguyen L (Nguyen et al., 2016)	—	Ⅰ	2016	77	single-arm	healthy nonsmoking male and female adult individuals (n = 77)	39 (18–55)
cabozantinib	Brose MS (Brose et al., 2022)	NCT03690388	Ⅲ	2022	258	double-arm	previously treated radioiodine-refractory differentiated thyroid cancer (n = 258)	65 (31–85)
cabozantinib	Choy E (Choy et al., 2022)	NCT01588821	Ⅱ	2022	37	single-arm	renal cell (n = 7), lung non-small (n = 5), osteosarcoma (n = 3), radioiodine-refractory differentiated thyroid cancer (n = 3), Ewing’s sarcoma (n = 3), chondrosarcoma (n = 2), leiomyosarcoma (n = 2), melanoma (n = 2), alveolar soft parts sarcoma (n = 1), head and neck squamous cell carcinoma (n = 1), adenoid cystic carcinoma (n = 1), chondroblastoma (n = 1), chordoma (n = 1), fibroblastic sarcoma (n = 1), liposarcoma (n = 1), myxofibrosarcoma (n = 1), salivary duct carcinoma (n = 1), olfactory neuroblastoma (n = 1)	54 (18–83)
cabozantinib	Nakaigawa N (Nakaigawa et al., 2023)	NCT03339219	Ⅱ	2023	35	single-arm	advanced renal cell carcinoma (n = 35)	63 (42–84)
cabozantinib	Procopio G (Procopio et al., 2023)	NCT03463681	Ⅱ	2023	31	single-arm	mRCC (n = 31)	62 (29–79)
pexidartinib	Smith CC (Smith et al., 2020)	NCT01349049	Ⅰ/Ⅱ	2020	90	single-arm	R/R FLT3-ITD-mutant AML (n = 90)	55.9 (22–83)
pexidartinib	Tap WD (Tap et al., 2019)	NCT02371369	Ⅲ	2021	61	double-arm	advanced tenosynovial giant cell tumor (n = 61)	44 (22–75)
pexidartinib	Lee JH (Lee et al., 2020)	NCT02734433	Ⅰ	2020	11	single-arm	bladder cancer/urothelial carcinoma (n = 1), epithelioid trophoblastic tumor (n = 1), gallbladder neuroendocrine carcinoma/large cell type (n = 1), liver cancer (n = 1), malignant fibrous histiocytoma (n = 1), renal cell carcinoma (n = 1), renal pelvic cancer, right; urothelial carcinoma (n = 1), sacral chordoma (n = 1), salivary gland cancer/right submandibular pleiomorphic adenocarcinoma (n = 1), submandibular gland/left; adenoid cystic carcinoma (n = 1), tenosynovial giant cell tumor (n = 1)	64 (23–82)
pexidartinib	Boal LH (Boal et al., 2020)	NCT02390752	Ⅰ	2020	16	single-arm	sarcomas (osteosarcoma, Ewing Sarcoma, rhabdomyosarcoma, malignant peripheral nerve sheath tumor) (n = 8), neurofibromatosis type 1 (NF1) plexiform neurofibroma (n = 3), central nervous system tumors (n = 3), AML (n = 1), peritoneal mesothelioma (n = 1)	16 (4–21)
pacritinib	Mesa RA (Mesa et al., 2017)	NCT01773187	Ⅲ	2017	220	double-arm	primary myelofibrosis (n = 144), post-polycythemia vera myelofibrosis (n = 48), post-essential thrombocythemia myelofibrosis (n = 27), missing (n = 1)	67 (60–73)
pacritinib	Verstovsek S (Verstovsek et al., 2016)	NCT00719836	Ⅰ/Ⅱ	2016	76	single-arm	myelofibrosis (n = 69), AML (n = 7)	69 (47–86)
pacritinib	Younes A (Younes et al., 2012)	NCT00741871	Ⅰ	2012	34	single-arm	advanced lymphoid malignancies (n = 34)	49 (22–80)
pacritinib	Komrokji RS (Komrokji et al., 2015)	NCT00745550	Ⅱ	2015	35	single-arm	primary and secondary myelofibrosis (n = 35)	69 (44–84)
TAK-659	Gordon LI (Gordon et al., 2020)	NCT02000934	Ⅰ	2020	105	single-arm	lymphomas (n = 86), solid tumors (n = 19)	65 (23–85)
TAK-659	Kaplan JB (Kaplan et al., 2016)	—	Ⅰ	2016	36	single-arm	diffuse large B-cell lymphoma (n = 30), follicular lymphoma (n = 4), mantle cell lymphoma (n = 1), chronic lymphocytic leukemia (n = 1)	60.5 (23–82)
TAK-659	Pratz KW (Pratz et al., 2023)	NCT02323113	Ⅰb	2023	43	single-arm	R/R AML (n = 43)	65 (25–86)
famitinib	Xu RH (Xu et al., 2017)	NCT01762293	Ⅱ	2017	99	double-arm	refractory metastatic colorectal cancer (n = 99)	55 (24–70)
famitinib	Zhang W (Zhang et al., 2013)	NCT01829841	Ⅰ/Ⅱ	2013	24	single-arm	mRCC (n = 24)	52 (24–66)
famitinib	Zhou A (Zhou et al., 2013)	—	Ⅰ	2013	55	single-arm	RCC (n = 12), sarcoma (n = 11), colorectal cancer (n = 7), lung cancer (n = 4), gastric cancer (n = 3), hepatocellular carcinoma (n = 2), breast cancer (n = 2), GIST (n = 2), nasopharyngeal carcinoma (n = 2), other (n = 10)	45 (19–67)

Note: AML, acute myeloid leukemia; R/R, relapsed/refractory; ITD, internal tandem duplication; TKD, tyrosine kinase domain; mRCC, metastatic renal cell carcinoma; HCC, hepatocellular carcinoma.

### Quality assessment

Considering that the included studies contained both randomized and non-randomized experiments, we assessed the quality of randomized and non-randomized studies using REVMAN and MINORS, respectively. As shown in Supplementary Table S1 and Supplementary Figure S2, the results of quality assessment showed that all studies received satisfactory scores.

## Safety

### Toxicity

**FIGURE 2:**
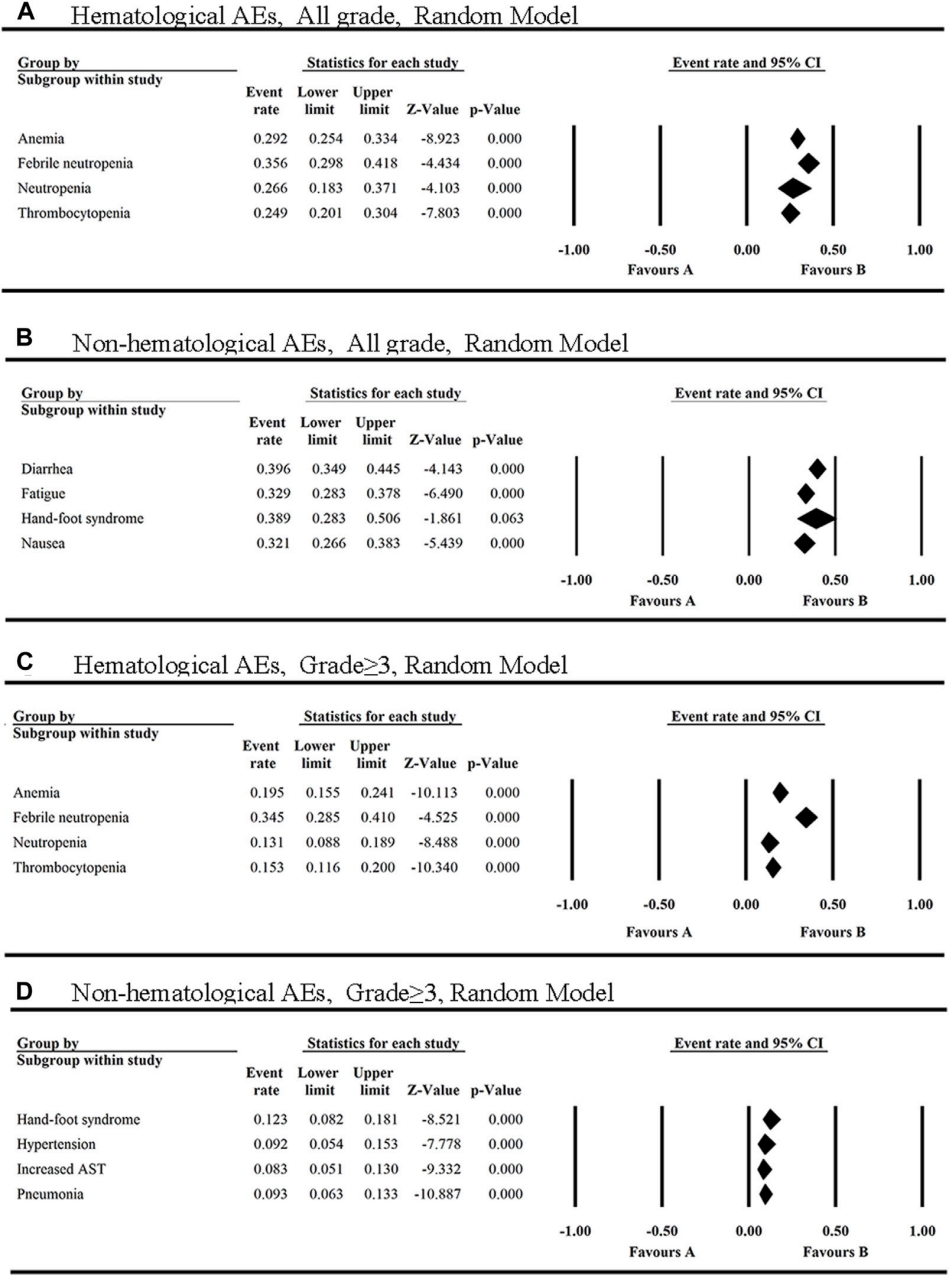
The top four hematological and non-hematological AEs of all grades **(A,B)** and grade ≥3 **(C,D)** in FLT3 inhibitors monotherapy.

The single-arm or double-arm studies with AEs of FLT3 inhibitors were selected in this study. Several clinical trials of FLT3 inhibitors were conducted in patients with hematological malignancies (leukemia, lymphoma, myelofibrosis) and solid tumors (glioblastoma, renal cancer, pancreatic cancer, bile duct cancer, lung cancer, colorectal cancer, head and neck cancer, soft tissue sarcoma, hepatocellular cancer, thyroid cancer, gastric cancer, prostate cancer, breast cancer, testicular cancer, mesothelioma, et al.). AEs are shown for all of these 13 FLT3 inhibitors. The top four AEs of all grades caused by the 12 FLT3 inhibitors were diarrhea, hand-foot syndrome, febrile neutropenia and fatigue (Figure 2). The top two hematological AEs of all grades and grade ≥3 caused by these FLT3 inhibitors were febrile neutropenia (35.6%, 34.5%) and anemia (29.2%, 19.5%) (Figures 2A, C). In addition, the top three all-grade non-hematologic AEs for the monotherapy with these FLT3 inhibitors were diarrhea (39.6%, 95% CI: 0.349–0.445), hand-foot syndrome (38.9%, 95% CI: 0.283–0.506) and fatigue (32.9%, 95% CI: 0.283–0.378) (Figure 2B).

Also, the most common AEs of all grades of FLT3 inhibitors in the non-hematological system were summarized. As shown in Supplementary Figure S3, the most common AEs of all grades occurred in gastrointestinal system (28.0%, 95% CI: 0.254–0.308), skin (26.2%, 95% CI: 0.205–0.329) and general disorders (24.0%, 95% CI: 0.214–0.267). In addition to diarrhea, the non-hematologic AEs of all grades with an overall incidence greater than 30% were hand-foot syndrome (38.9%, 95% CI: 0.283–0.506), fatigue (32.9%, 95% CI: 0.283–0.378) and nausea (32.1%, 95% CI: 0.266–0.383) (Supplementary Figure S4).

However, the top three grade ≥3 non-hematologic AEs among the 12 FLT3 inhibitors were hand-foot syndrome (HFS) (12.3%), pneumonia (9.3%), and hypertension (9.2%) (Figure 2D). Additionally, the grade≥3 AEs of FLT3 inhibitors with the incidence ≥5% have increased AST, pyrexia, hypokalaemia, increased ALT, fatigue and diarrhea (Supplementary Figure S5).

Febrile neutropenia was reported in 14 articles with eight FLT3 inhibitors, including sorafenib, midostaurin, gilteritinib, quizartinib, cabozantinib, pexidartinib, tandutinib and TAK-659 (Supplementary Figure S6A), and ranked first in all grades and grade≥3 of hematologic AEs. The incidence of febrile neutropenia of all grades ranged from 10.8% (quizartinib) to 60.0% (TAK-659) with the average of 35.6% (95% CI: 0.298, 0.418). Moreover, febrile neutropenia caused by FLT3 inhibitors was mainly in grade≥3 [34.5% (95% CI: 0.285, 0.410)] (Figure 2).

Diarrhea was the most common AE of all grades reported in all of these 13 FLT3 inhibitors and fourty-two of the clinical trials included in this study (Supplementary Figure S6B). The incidence of diarrhea in these inhibitors was all greater than 20%, with the highest incidence of 64.4% caused by cabozantinib, and the lowest of 21.8% by famitinib.

### Dose-limiting toxicity (DLT)

The DLT was found in the monotherapy of 11 FLT3 inhibitors, including cabozantinib, gilteritinib, lestaurtinib, midostaurin, pacritinib, pexidartinib, quizartinib, sorafenib, sunitinib, TAK-659 and tandutinib (Supplementary Table S2). The known DLTs were diarrhea, nausea, vomiting, fatigue, QT prolongation, anemia, thrombocytopenia, hand–foot syndrome, hypertension, edema, pain, weight loss, anorexia, dyspepsia, asthenia, dehydration, tumor lysis syndrome, syncope, elevated amylase, elevated blood creatine phosphokinase, elevated blood lactate dehydrogenase, increased lipase, increased glutamyltransferase, increased aspartate aminotransferase, hypoxia, proteinuria, pancreatitis, transaminitis, stomatitis, mucositis, gastrointestinal bleeding, dizziness, hypophosphatemia. Among them, the most common DLTs were diarrhea, nausea, fatigue, and QT prolongation. Therefore, the dosing regimen and dosage should be timely adjusted according to the patient’s condition. In addition, we paid special attention to the IC50 of FLT3 inhibitors in tumors and the final human doses (Supplementary Tables S3, S4).

### Emergency AEs leading to drug withdrawal

Some AEs can be life-threatening to the patient: The fatal AEs associated with pexidartinib is cytokine release syndrome, sepsis, pneumonia, pneumonia aspiration, respiratory failure, cardiac arrest and cerebral hemorrhage, which twelve patients died of reported by Smith CC et al (Smith et al., 2020) The fatal AEs caused by gilteritinib were pneumonia, subdural hematoma, elevated aspartate aminotransferase, elevated alanine aminotransferase, elevated blood creatine phosphokinase and elevated lactate dehydrogenase (Usuki et al., 2018; Perl et al., 2019), while the most common emergency AEs of quizartinib leading to discontinuation or death was QT prolongation, pneumonia, sepsis, pericardial effusion, pericarditis, diarrhea, neutropenic sepsis, pleural effusion, intracranial haemorrhage, bronchopulmonary aspergillosis (Cortes J. et al., 2018; Cortes JE. et al., 2018; Cortes et al., 2019; Usuki et al., 2019). For pacritinib, the patients discontinued treatment due to diarrhea, QT prolongation, fatigue, increased transaminases, hypersensitivity, pruritus, thrombocytopenia, hyperbilirubinemia, subdural hematoma, and nausea, while the fatal AEs were pneumonia, subdural hematoma, intracranial hemorrhage, septic shock, asthenia, cardiorespiratory arrest, anemia, subdural hematoma and AML (Komrokji et al., 2015; Verstovsek et al., 2016; Mesa et al., 2017). For TAK-659, the drug-related serious AEs leading to discontinuation were sepsis and pneumonia, and those leading to death were sepsis (Kaplan et al., 2016) and multiorgan failure (Pratz et al., 2023).

To compare the toxicity of different drugs, we collected the percentage of patients discontinuing due to severe AEs caused by these FLT3 inhibitors reported in the included articles (Supplementary Figure S7). A total of 32 articles reported the percentage of patients discontinuing due to severe AEs of 12 FLT3 inhibitors. The overall incidence was 16.4% (95% CI: 0.137–0.195), the lowest was caused by lestaurtinib (6.9%), and the highest was by sorafenib (23.5%). This data indicated that sorafenib had the highest toxicity among these drugs.

### Pharmacokinetics

Based on published pharmacokinetic results (Table 2), the *T*_max_ for these FLT3 inhibitors ranged from 0.7–12.0 h. Among them, the FLT3 inhibitor with the shortest *T*_max_ is midostaurin and lestaurtinib, while the one with the longest *T*_max_ is quizartinib. The published *T*_*1/2*_ for most of the FLT3 inhibitors exceeded 10 h. The longest *T*_*1/2*_ was 84.0–146.0 h, 84.0–126.0 h and 107.8 h for cabozantinib, giltertinib and quizartinib, respectively, and the shortest was 6.8–9.2 h for lestaurtinib. This suggested that cabozantinib, giltertinib and quizartinib may have the longest duration of action, while lestaurtinib may have the shortest.

**TABLE 2:** Pharmacodynamics of 13 FLT3 inhibitors.

FLT3 inhibitors	Author/publication year	*T*_*max*_	*T*_*1/2*_
sorafenib	Huh KY, 2021 (Huh et al., 2021)	4.0 h	22.2 ± 5.1 h
sorafenib	Awada A, 2005 (Awada et al., 2005)	-	24.0–39.0 h
lestaurtinib	Smith BD, 2004 (Smith et al., 2004)	-	6.8–9.2 h
lestaurtinib	Marshall JL, 2005 (Marshall et al., 2005)	0.8–2.7 h	-
midostaurin	Propper DJ, 2001 (Propper et al., 2001)	-	38.4 h
midostaurin	He H, 2017 (He et al., 2017)	0.7–2.7 h	20.3 ± 6.7 h
gilteritinib	Numan Y, 2022 (Numan et al., 2022)	3.0–7.0	84.0–126 h
gilteritinib	Perl AE, 2017 (Perl et al., 2017)	2.0–6.1 h	45.9–151.8 h
quizartinib	Usuki K, 2019 (Usuki et al., 2019)	5.24 h	-
quizartinib	Li J, 2020 (Li et al., 2020)	4.0–12.0 h	107.8 h
sunitinib	DuBois SG, 2012 (DuBois et al., 2012)	4.0–8.0 h	-
sunitinib	Britten CD, 2008 (Britten et al., 2008)	6.0 h	-
sunitinib	O'Farrell AM, 2003 (O'Farrell et al., 2003)	4.0–8.0 h	44.0 ± 18.6 h
sunitinib	Faivre S, 2006 (Faivre et al., 2006)	5.0 h	41.0–86.0 h
crenolanib	Lewis NL, 2009 (Lewis et al., 2009)	4.0–6.0 h	12.3–18.5 h
tandutinib	Grossman SA, 2017 (NCT00379080, 2017)	-	10.4–13.2 h
cabozantinib	Nguyen L, 2016 (Brose et al., 2022)	3.5–4.0 h	84.0–146.0 h
pexidartinib	Lee JH, 2020 (Lee et al., 2020)	1.0–2.1 h	-
pexidartinib	Boal LH, 2020 (Boal et al., 2020)	2.0–12 h	12.7–24.2 h
pacritinib	Younes A, 2012 (Younes et al., 2012)	5.0–9.0 h	24.0–96.0 h
TAK-659	Kaplan JB, 2016 (Kaplan et al., 2016)	2.0–3.0 h	-
TAK-659	Pratz KW, 2023 (Pratz et al., 2023)	1.0–3.0 h	-
famitinib	Zhou A, 2013 (Zhou et al., 2013)	3.3–5.3 h	28.7–33.8 h

## Efficacy

### Survival outcome

**FIGURE 3:**
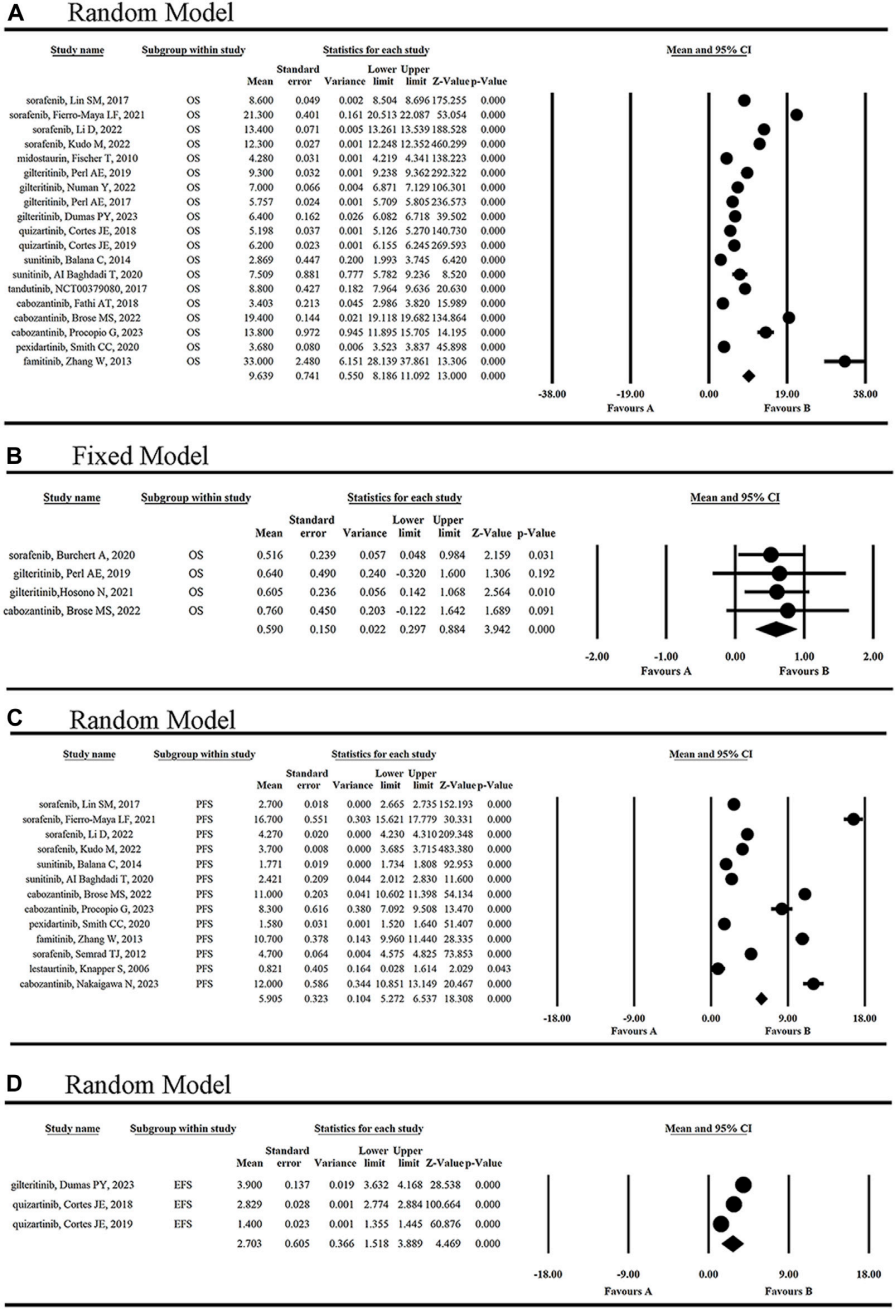
The statistical analysis results of OR of single-arm **(A)** and double-arm **(B)** studies, and of PFS **(C)** and EFS **(D)** of FLT3 inhibitors.

As shown in Figure 2A, nine FLT3 inhibitors included in a total of 17 clinical trials prolonged overall survival in tumor patients, with the mean OS of 9.639 months (Figure 3A). When one of sorafenib, gilteritinib and cabozantinib was used to treat FLT3-mutated AML or radioiodine-refractory differentiated thyroid cancer, the mean HR for death was 59.0% (95% CI 0.297, 0.884) (Figure 3B). Among them, the strongest anti-tumor effect was achieved by sorafenib [HR 0.516 (95% CI: 0.048, 0.984, *p* = 0.031)]. However, cabozantinib [HR 2.27 (95% CI: 1.030, 5.004, *p* = 0.042)] was not recommended for the treatment of recurrent ovarian cancer at the doses and schedule studied in this study (Matulonis et al., 2019).

As shown in Figure 3C, six FLT3 inhibitors sorafenib, sunitinib, cabozantinib, pexidartinib, famitinib and lestaurtinib, prolonged mPFS of 5.905 months (95% CI 5.272, 6.537). EFS was reported in three articles for two inhibitors, gilteritinib and quizartinib, and the mEFS was 2.703 months (95% CI 1.518, 3.889) (Figure 3D). Simultaneously, duration of therapy was reported in sorafenib, gilteritinib and quizartinib with the range of 1.94–4.6 months (Awada et al., 2005; Borthakur et al., 2011; Semrad et al., 2012; Chen et al., 2014; Lin et al., 2017; Perl et al., 2017; Cortes J. et al., 2018; Cortes JE. et al., 2018; Usuki et al., 2018; Cortes et al., 2019; Perl et al., 2019; Usuki et al., 2019; Burchert et al., 2020; Li et al., 2020; Fierro-Maya et al., 2021; Hosono et al., 2021; Huh et al., 2021; Li et al., 2022; Numan et al., 2022; Dumas et al., 2023; Kudo et al., 2023).

### Response outcomes

**FIGURE 4:**
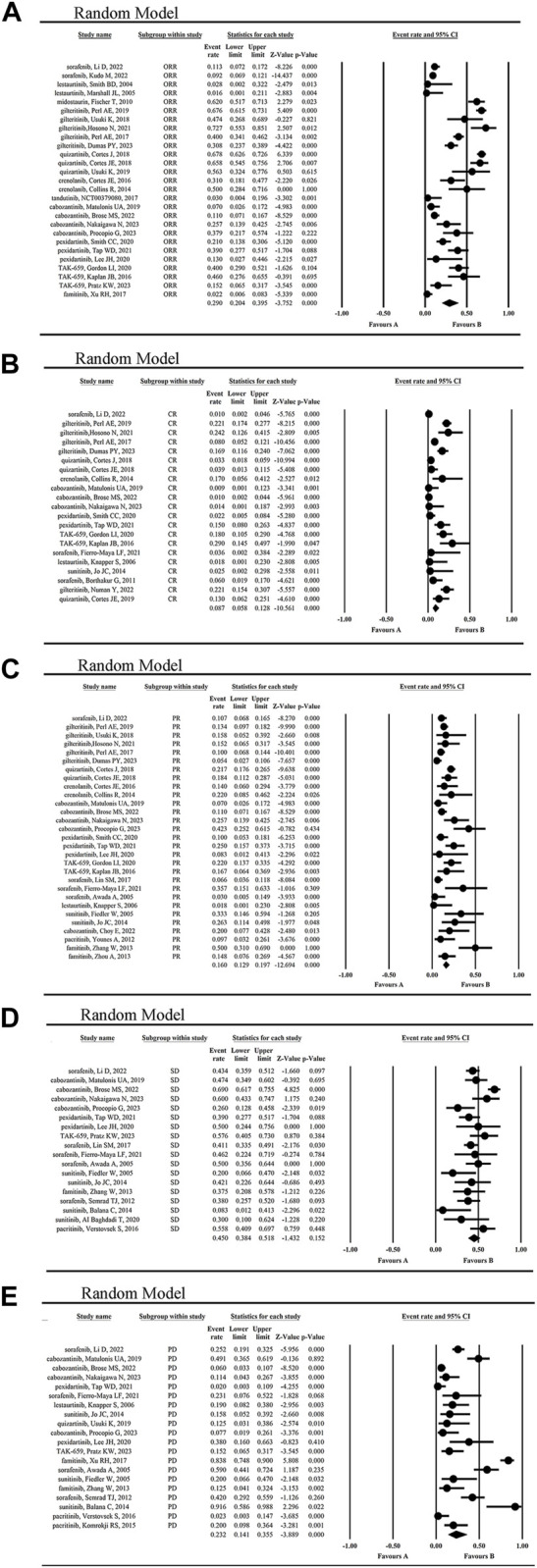
The statistical analysis results of ORR **(A)**, CR **(B)**, PR **(C)**, SD **(D)** and PD **(E)** of FLT3 inhibitors.

The mean ORR for tumor patients was 29.0% (95% CI 0.204, 0.395) (Figure 4A). The highest ORR of 72.7% was achieved by giltertinib in the treatment of FLT3-ITD mutated AML, while the lowest of 1.6% and 2.8% occurred in the treatment of the solid tumors and AML by lestaurtinib (Marshall et al., 2005; Knapper et al., 2006), respectively, indicating the poor efficacy of lestaurtinib.

The mean CR rate of patients was 8.7% (95% CI 0.058, 0.128) (Figure 4B), while the highest CR rate was 29.0% reported by Kaplan JB when using TAK-659 to treat lymphoma and leukemia (Kaplan et al., 2016). However, the lowest CR rate of 0.9%–1.4% occurred in the cabozantinib treatment group of solid tumors (Matulonis et al., 2019; Brose et al., 2022; Nakaigawa et al., 2023).

The mean PR rate reported for patients was 16.0% (95% CI, 0.129, 0.197) (Figure 4C), with a maximum PR rate of 50% when using famitinib to treat metastatic renal cell carcinoma (mRCC) (Zhang et al., 2013), and a minimum of 7% in the treatment of gynecological tumors using cabozantinib (Matulonis et al., 2019). The second highest PR rate was 42.3%, which was achieved with cabozantinib in the treatment of mRCC (Procopio et al., 2023).

The mean SD rate for tumor patients was 45.0% (95% CI 0.384, 0.518) (Figure 4D), with a maximum SD rate of 69% in the treatment of advanced RCC using cabozantinib (Brose et al., 2022), and a minimum of 8.3% in the treatment of non-resectable glioblastoma using sunitinib (Balaña et al., 2014). In contrast, the mean PD rate for tumor patients was 23.2% (95% CI 0.141, 0.355) (Figure 4E), with a maximum PD rate of 91.6% in the treatment of non-resectable glioblastoma using sunitinib (Balaña et al., 2014), and a minimum of 2.3% in the treatment of myelofibrosis and AML using pacritinib (Verstovsek et al., 2016). From these results, we concluded that sunitinib was not so effective in the treatment of primary glioblastoma.

**FIGURE 5:**
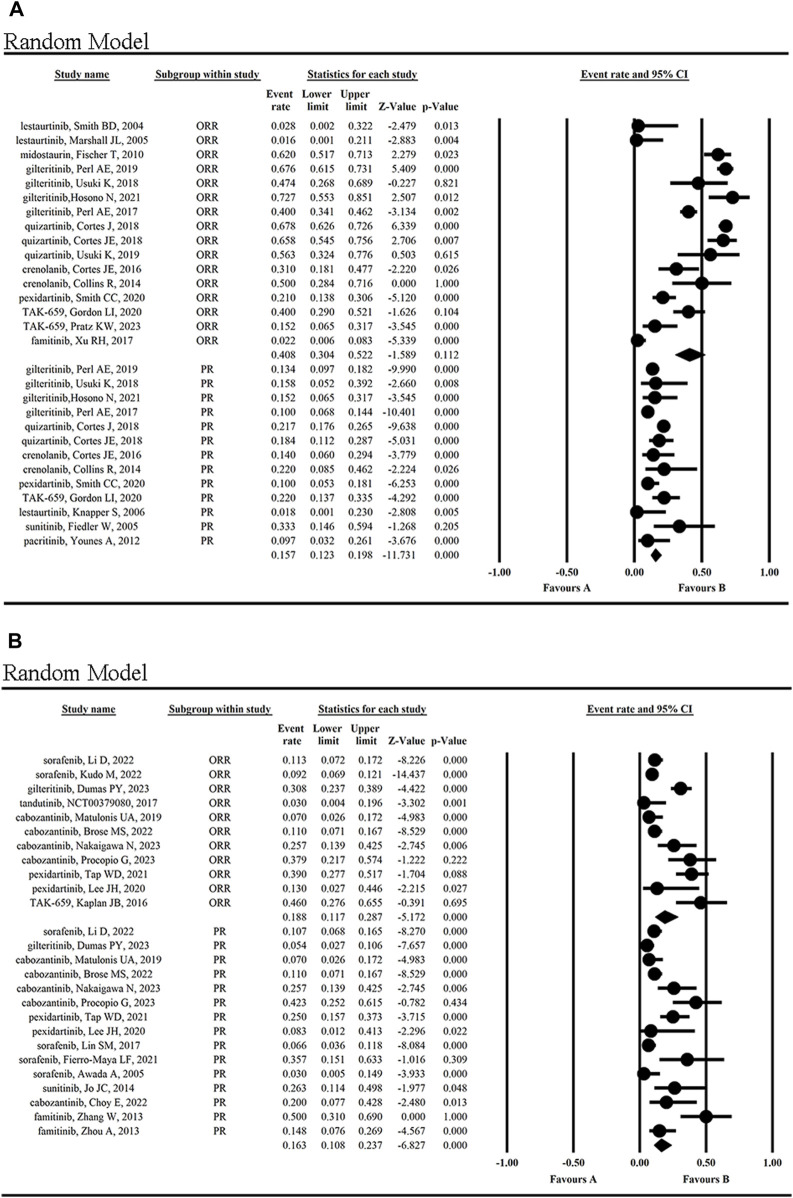
Clinical effects (ORR and PR) of FLT3 inhibitors in hematological malignancies **(A)** and solid tumors **(B)**.

Additionally, the clinical effect of FLT3 inhibitors in hematologic malignancies and solid tumors was compared. The statistical results showed the ORR (40.8% vs. 18.8%) and CR (10.3% vs. 2.3%) were higher in hematological malignancies than in solid tumors, while the PR (15.7% vs. 16.3%) and SD (47.5% vs. 44.4%) was not significantly different from each other (Figure 5; Supplementary Figure S8). The statistical results showed the OS (5.694 months vs. 13.343 months) was lower in hematological malignancies than in solid tumors (Supplementary Figure S9).

### Target genes

The target genes of these FLT3 inhibitors in the published articles were summarized in the Supplementary Table S5. Among them, their common target gene was FLT3, and the other core genes regulated by these 13 FLT3 inhibitors were mainly AKT1, KIT, MTOR, PDGFR, STAT5 and STAT3 (Supplementary Table S6).

## Discussion

FLT3 is one of type III receptor tyrosine kinases that plays an important role in cell survival, proliferation and differentiation. FLT3 mutations are the most common genetic aberrations in acute myeloid leukemia (AML): approximately 25% of adult patients with AML carry FLT3-ITD mutation and 10% carry FLT3-TKD point mutations or deletions (Kiyoi et al., 2020). Both mutant FLT3 molecules are activated through ligand-independent dimerization and trans-phosphorylation, resulting in constitutive activation (Kiyoi et al., 2020). Mutant FLT3 induces the activation of multiple intracellular signaling pathways, mainly STAT5, MAPK and AKT signals, leading to cell proliferation and anti-apoptosis effect.

The patients with *FLT3*-mutated AML have a poor prognosis compared to those with FLT3-WT (wild-type). Though response rates to traditional chemotherapy are similar in FLT3-mutated AML compared to FLT3- AML, *FLT3*-mutated AML patients are more likely to relapse, even after allogeneic hematopoietic stem-cell transplantation (HSCT) (Schlenk et al., 2008; Bazarbachi et al., 2020). Therefore, the advent of targeted FLT3 inhibitors widens treatment options in these FLT3-mutated patients.

The type I inhibitors including lestaurtinib, sunitinib, midostaurin, crenolanib, gilteritinib, cabozantinib, pexidartinib, pacritinib, TAK-659 and famitinib, can bind to both active and inactive conformations of FLT3. Except FLT3, these inhibitors can still bind to the other kinases which share the similar protein structure of the ATP-binding region with FLT3. However, the type II inhibitors including sorafenib, quizartinib and tandutinib, only can bind to an inactive conformation. They insert into the back pocket of the ATP-binding region of inactive FLT3, and interact with its amino acid residues, promoting inhibitory activity and selectivity. However, they have no binding affinity to an active conformation of FLT3, due to the use of the back pocket of the ATP-binding region.

The efficacy of FLT3 inhibitors through inducing cell apoptosis, ferroptosis, Pyroptosis, and/or differentiation (Hage et al., 2019; Arries and Yohe, 2020; Gao et al., 2021; Yuan et al., 2022) have been extensively proved in the various oncology indications. However, to date, the AEs and effects of these inhibitors have not been reported extensively and comprehensively. In this study, the published FLT3 inhibitors of type I and II are selected. Their safety, efficacy, pharmacodynamics and target genes are systematically analyzed based on registered clinical trials, published articles and public database.

With regards to the toxicity of these inhibitors, we analyzed AEs caused by FLT3 inhibitors monotherapy in patients with hematological diseases and solid tumors. The most common hematological AEs caused by these inhibitors were febrile neutropenia, anemia, and thrombocytopenia, and they were also the most severe hematological AEs. Febrile neutropenia of all grades was observed in approximately 35% of the patients. In addition, the most common non-hematologic AEs were diarrhea, hand-foot syndrome, fatigue, and nausea in descending order.

FLT3 inhibitors are non-specific, and can also suppress VEGFR, PDGFRA, PDGFRB, AXL, EGFR, and KIT. The inhibition of these receptors, which are also expressed on normal cells, by FLT3 inhibitors may lead to extrahematological toxicity, for example, cutaneous, gastrointestinal, and cardiovascular toxicities.

Notably, in clinic, the overall safety of TKIs as well as the incidence of the most common AEs (especially specific AEs) is considered firstly, rather than the efficacy (Krawczyk et al., 2023). The diverse emergency AEs, especially pneumonia, which led to drug discontinuation, even the death of the patients, was reported in 12 FLT3 inhibitors. Therefore, in the treatment of tumors using FLT3 inhibitors, the infection should be controlled timely to prevent the occurrence of fatal complications such as pneumonia, sepsis and respiratory failure.

Additionally, the DLTs of FLT3 inhibitors varied in different inhibitors, but the most common and highest occurring DLT was fatigue. Moreover, the available clinical data showed a significant increase in the incidence of AEs caused by these inhibitors compared to the placebo group. Therefore, it is necessary to closely monitor the extent of AEs and discontinue the medication if necessary.

Based on the current data, after monotherapy with thirteen FLT3 inhibitors, the patients’ OS, PFS, and EFS were 9.639, 5.905 and 2.703 months, respectively. Previous studies have indicated that the combination of ICIs and TKIs as first-line treatment can significantly improved OS in patients with advanced HCC and was associated with better PFS (Wu et al., 2023). Furthermore, the overall ORR, CR, PR, SD and PD were 29.0%, 8.7%, 16.0%, 45.0% and 23.2%, respectively, indicating the good efficacy of FLT3 inhibitors as a whole. The patient’s highest CR reached 29%, when TAK-659 was used to treat lymphoma and leukemia (Kaplan et al., 2016), and the overall ORR was higher in hematological malignancies than in solid tumors (40.8% vs. 18.8%), indicating FLT3 inhibitors might be more effective when applied in the treatment of hematological malignancies than in that of solid tumors. What’s more, although all of these inhibitors share the common target FLT3, lestaurtinib and cabozantinib had not shown satisfactory results, so it was not recommended in the treatment of hematological and/or solid malignancies.

The *T*_max_ for these FLT3 inhibitors was between 0.7 and 12.0 h, indicating rapid oral absorption efficiency. Take sorafenib for example, considering possibly decreased bioavailability under high-fat meal, sorafenib can be administered without food or with low/moderate-fat meal (Di Gion et al., 2011). In contrast, sunitinib and quizartinib can be administered without regard to food (Di Gion et al., 2011; Li et al., 2020), and the bioavailability of midostaurin and cabozantinib increases significantly under high-fat fed condition (Wang et al., 2008; Lacy et al., 2017). Additionally, pexidartinib is recommended to be administered with a low-fat meal (Zahir et al., 2023). As shown in Table 2, the *T*_*1/2*_ of FLT3 inhibitors varied significantly. The longest *T*_*1/2*_ was approximately 100 h for cabozantinib, giltertinib and quizartinib, while the shortest *T*_*1/2*_ were 6.8–9.2 h for lestaurtinib, which may well explain the reason why these FLT3 inhibitors have different clinical effects.

In short, thirteen FLT3 inhibitors were included and evaluated in this study. Although they had distinct pharmacodynamics profiles and clinical response data, all of them exhibited similar safety outcomes. Their overlapping toxicities were mainly diarrhea and febrile neutropenia, which were simultaneously the most common and severe AEs. In addition, cabozantinib and quizartinib showed a more favorable pharmacodynamics profile with a longer half-life of ≥100 h. By contrast, lestaurtinib had an unfavorable clinical pharmacodynamics profile with the shortest half-life. Based on the available data, except lestaurtinib and cabozantinib, the other FLT3 inhibitors showed obvious anti-tumor effects. The patients with different tumors benefited from different FLT3 inhibitors, and those with hematological malignancies benefited more than solid tumors. And FLT3 inhibitors can be used with or after chemotherapeutic agents (Awada et al., 2005). However, the emergency AEs caused by these inhibitors should be paid special attention to in the treatment of tumors.

### Study Highlights

#### What is the current knowledge on the topic?

Several FLT3 inhibitors (FLT3i) are in clinical trials. However, the safety, efficacy and pharmacodynamics of these FLT3i have not been systemically analyzed before.

#### What question did this study address?

In this study, we analyzed the published clinical trials and summarized the safety, efficacy and pharmacokinetics of FLT3i including sorafenib, lestaurtinib, midostaurin, gilteritinib, quizartinib, sunitinib, crenolanib, tandutinib, cabozantinib, pexidartinib, pacritinib, TAK-659 (mivavotinib) and famitinib.

#### What does this study add to our knowledge?

The most common adverse events (AEs) of FLT3i were gastrointestinal adverse reactions, including diarrhea, hand-foot syndrome and nausea, while the most common hematological AEs were febrile neutropenia, anemia, and thrombocytopenia. FLT3i monotherapy has shown significant anti-tumor effect in clinic, especially in hematologic malignancies.

#### How might this change clinical pharmacology or translational science?

FLT3i monotherapy has shown significant anti-tumor effect in clinic, which can be improved further through structural modification and combination medication. Meanwhile, the AEs of these FLT3i implied the safety should be closely monitored when used clinically.

### Limitations

Here, there are some inevitable factors to affect our systematic analysis results. First, most of the involved studies were single-armed, thus not designed according to the principle of randomized controlled trials. Secondly, the drugs were in the different phases of clinical trials, so the data adopted in this study were from phases I/II/III/IV. Thirdly, the data on AEs, efficacy, pharmacokinetic and pharmacodynamic analysis were not published completely, and these clinical studies enrolled the patients with different types and stages of tumors, which were administered the different drugs, doses and times, making a systemic, comprehensive retrospective comparison of antitumor clinical effect among these drugs not possible.

## Data Availability

The original contributions presented in the study are included in the article/Supplementary Material, further inquiries can be directed to the corresponding authors.

## Supplementary material

SUPPLEMENTARY FIGURE S1 Chemical formulae of 13 FLT3 inhibitors.

SUPPLEMENTARY FIGURE S2 The risk of study bias in randomized controlled trials was assessed using the Cochrane Risk of Bias tool.

SUPPLEMENTARY FIGURE S3 Results of AEs of all grades occurring in different body systems.

SUPPLEMENTARY FIGURE S4 Results of non-hematological AEs of all-grades.

SUPPLEMENTARY FIGURE S5 Results of non-hematological grade ≥3 AEs.

SUPPLEMENTARY FIGURE S6 Incidence of all-grade febrile neutropenia and diarrhea.

SUPPLEMENTARY FIGURE S7 Percentage of patients of discontinuation of medication due to AEs caused by FLT3 inhibitors.

SUPPLEMENTARY FIGURE S8 Clinical effects (CR,SD and PD) of FLT3 inhibitors in hematological malignancies **(A)** and solid tumors **(B)**.

SUPPLEMENTARY FIGURE S9 Clinical effects (OS) of FLT3 inhibitors in hematological malignancies **(A)** and solid tumors **(B)**.

## Abbreviations

AML, acute myeloid leukemia; AE, adverse event; OS, overall survival; PFS, progression-free survival; CR, complete remission; ORR, overall response rate; PR, partial response; SD, stable disease.

## References

[B1] Al B. T. Garrett-Mayer E. Halabi S. Mangat P. K. Rich P. Ahn E. R. (2020). Sunitinib in patients with metastatic colorectal cancer (mCRC) with FLT-3 amplification: results from the targeted agent and profiling utilization registry (TAPUR) study. Target Oncol. 15 (6), 743–750. 10.1007/s11523-020-00752-8 33068284

[B2] Arries C. D. Yohe S. L. (2020). Monocytic maturation induced by FLT3 inhibitor therapy of acute myeloid leukemia: morphologic and immunophenotypic characteristics. Lab. Med. 51 (5), 478–483. 10.1093/labmed/lmz094 31872224

[B3] Awada A. Hendlisz A. Gil T. Bartholomeus S. Mano M. de Valeriola D. (2005). Phase I safety and pharmacokinetics of BAY 43-9006 administered for 21 days on/7 days off in patients with advanced, refractory solid tumours. Br. J. Cancer 92 (10), 1855–1861. 10.1038/sj.bjc.6602584 15870716 PMC2361774

[B4] Balaña C. Gil M. J. Perez P. Reynes G. Gallego O. Ribalta T. (2014). Sunitinib administered prior to radiotherapy in patients with non-resectable glioblastoma: results of a phase II study. Target Oncol. 9 (4), 321–329. 10.1007/s11523-014-0305-1 24424564

[B5] Bazarbachi A. Bug G. Baron F. Brissot E. Ciceri F. Dalle I. A. (2020). Clinical practice recommendation on hematopoietic stem cell transplantation for acute myeloid leukemia patients with FLT3-internal tandem duplication: a position statement from the Acute Leukemia Working Party of the European Society for Blood and Marrow Transplantation. Haematologica 105 (6), 1507–1516. 10.3324/haematol.2019.243410 32241850 PMC7271578

[B6] Boal L. H. Glod J. Spencer M. Kasai M. Derdak J. Dombi E. (2020). Pediatric PK/PD phase I trial of pexidartinib in relapsed and refractory leukemias and solid tumors including neurofibromatosis type I-related plexiform neurofibromas. Clin. Cancer Res. 26 (23), 6112–6121. 10.1158/1078-0432.CCR-20-1696 32943455 PMC7909006

[B7] Borthakur G. Kantarjian H. Ravandi F. Zhang W. Konopleva M. Wright J. J. (2011). Phase I study of sorafenib in patients with refractory or relapsed acute leukemias. Haematologica 96 (1), 62–68. 10.3324/haematol.2010.030452 20952518 PMC3012766

[B8] Britten C. D. Kabbinavar F. Hecht J. R. Bello C. L. Li J. Baum C. (2008). A phase I and pharmacokinetic study of sunitinib administered daily for 2 weeks, followed by a 1-week off period. Cancer Chemother. Pharmacol. 61 (3), 515–524. 10.1007/s00280-007-0498-4 17505827

[B9] Brose M. S. Robinson B. G. Sherman S. I. Jarzab B. Lin C. C. Vaisman F. (2022). Cabozantinib for previously treated radioiodine-refractory differentiated thyroid cancer: updated results from the phase 3 COSMIC-311 trial. Cancer 128 (24), 4203–4212. 10.1002/cncr.34493 36259380 PMC10092751

[B10] Burchert A. Bug G. Fritz L. V. Finke J. Stelljes M. Röllig C. (2020). Sorafenib maintenance after allogeneic hematopoietic stem cell transplantation for acute myeloid leukemia with *FLT3*-internal tandem duplication mutation (SORMAIN). J. Clin. Oncol. 38 (26), 2993–3002. 10.1200/JCO.19.03345 32673171

[B11] Chen Y. B. Li S. Lane A. A. Connolly C. Del Rio C. Valles B. (2014). Phase I trial of maintenance sorafenib after allogeneic hematopoietic stem cell transplantation for fms-like tyrosine kinase 3 internal tandem duplication acute myeloid leukemia. Biol. Blood Marrow Transpl. 20 (12), 2042–2048. 10.1016/j.bbmt.2014.09.007 PMC4253683 25239228

[B12] Choy E. Cote G. M. Michaelson M. D. Wirth L. Gainor J. F. Muzikansky A. (2022). Phase II study of cabozantinib in patients with bone metastasis. Oncologist 27 (7), 600–606. 10.1093/oncolo/oyac083 35524758 PMC9256024

[B13] Collins R. Kantarjian H. M. Levis M. J. Perl A. E. Ramachandran A. Ravandi F. (2014). Clinical activity of Crenolanib in patients with D835 mutant FLT3-positive relapsed/refractory acute myeloid leukemia (AML). J. Clin. Oncol. 32 (15_Suppl. l), 7027. 10.1200/jco.2014.32.15_suppl.7027

[B14] Cortes J. Perl A. E. Döhner H. Kantarjian H. Martinelli G. Kovacsovics T. (2018a). Quizartinib, an FLT3 inhibitor, as monotherapy in patients with relapsed or refractory acute myeloid leukaemia: an open-label, multicentre, single-arm, phase 2 trial. Lancet Oncol. 19 (7), 889–903. 10.1016/S1470-2045(18)30240-7 29859851 PMC8152787

[B15] Cortes J. E. Kantarjian H. M. Kadia T. M. Borthakur G. Konopleva M. Garcia-Manero G. (2016). Crenolanib besylate, a type I pan-FLT3 inhibitor, to demonstrate clinical activity in multiply relapsed FLT3-ITD and D835 AML. J. Clin. Oncol. 34 (15_Suppl. l), 7008. 10.1200/jco.2016.34.15_suppl.7008

[B16] Cortes J. E. Khaled S. Martinelli G. Perl A. E. Ganguly S. Russell N. (2019). Quizartinib versus salvage chemotherapy in relapsed or refractory FLT3-ITD acute myeloid leukaemia (QuANTUM-R): a multicentre, randomised, controlled, open-label, phase 3 trial. Lancet Oncol. 20 (7), 984–997. 10.1016/S1470-2045(19)30150-0 31175001

[B17] Cortes J. E. Tallman M. S. Schiller G. J. Trone D. Gammon G. Goldberg S. L. (2018b). Phase 2b study of 2 dosing regimens of quizartinib monotherapy in *FLT3*-ITD–mutated, relapsed or refractory AML. Blood 132 (6), 598–607. 10.1182/blood-2018-01-821629 29875101 PMC6085992

[B18] DeAngelo D. J. Stone R. M. Heaney M. L. Nimer S. D. Paquette R. L. Klisovic R. B. (2006). Phase 1 clinical results with tandutinib (MLN518), a novel FLT3 antagonist, in patients with acute myelogenous leukemia or high-risk myelodysplastic syndrome: safety, pharmacokinetics, and pharmacodynamics. Blood 108 (12), 3674–3681. 10.1182/blood-2006-02-005702 16902153 PMC1895460

[B19] Di Gion P. Kanefendt F. Lindauer A. Scheffler M. Doroshyenko O. Fuhr U. (2011). Clinical pharmacokinetics of tyrosine kinase inhibitors: focus on pyrimidines, pyridines and pyrroles. Clin. Pharmacokinet. 50 (9), 551–603. 10.2165/11593320-000000000-00000 21827214

[B20] DuBois S. G. Shusterman S. Reid J. M. Ingle A. M. Ahern C. H. Baruchel S. (2012). Tolerability and pharmacokinetic profile of a sunitinib powder formulation in pediatric patients with refractory solid tumors: a Children's Oncology Group study. Cancer Chemother. Pharmacol. 69 (4), 1021–1027. 10.1007/s00280-011-1798-2 22179104 PMC4008320

[B21] Dumas P. Y. Raffoux E. Bérard E. Bertoli S. Hospital M. A. Heiblig M. (2023). Gilteritinib activity in refractory or relapsed FLT3-mutated acute myeloid leukemia patients previously treated by intensive chemotherapy and midostaurin: a study from the French AML Intergroup ALFA/FILO. Leukemia 37 (1), 91–101. 10.1038/s41375-022-01742-7 36376378

[B22] Escudier B. Eisen T. Stadler W. M. Szczylik C. Oudard S. Siebels M. (2007). Sorafenib in advanced clear-cell renal-cell carcinoma. N. Engl. J. Med. 356, 125–134. 10.1056/NEJMoa060655 17215530

[B23] Faivre S. Delbaldo C. Vera K. Robert C. Lozahic S. Lassau N. (2006). Safety, pharmacokinetic, and antitumor activity of SU11248, a novel oral multitarget tyrosine kinase inhibitor, in patients with cancer. J. Clin. Oncol. 24 (1), 25–35. 10.1200/JCO.2005.02.2194 16314617

[B24] Fathi A. T. Blonquist T. M. Hernandez D. Amrein P. C. Ballen K. K. McMasters M. (2018). Cabozantinib is well tolerated in acute myeloid leukemia and effectively inhibits the resistance-conferring FLT3/tyrosine kinase domain/F691 mutation. Cancer 124 (2), 306–314. 10.1002/cncr.31038 28960265 PMC8167813

[B25] Fiedler W. Serve H. Döhner H. Schwittay M. Ottmann O. G. O'Farrell A. M. (2005). A phase 1 study of SU11248 in the treatment of patients with refractory or resistant acute myeloid leukemia (AML) or not amenable to conventional therapy for the disease. Blood 105 (3), 986–993. 10.1182/blood-2004-05-1846 15459012

[B26] Fierro-Maya L. F. González G. G. Melo L. J. R. Cuéllar A. A. C. Carreño A. Córdoba C. (2021). Safety and efficacy of sorafenib in patients with advanced thyroid carcinoma: a phase II study (NCT02084732). Arch. Endocrinol. Metab. 27, 2359–3997000000357. 10.20945/2359-3997000000373 PMC10522190 39421700

[B27] Fischer T. Stone R. M. Deangelo D. J. Galinsky I. Estey E. Lanza C. (2010). Phase IIB trial of oral Midostaurin (PKC412), the FMS-like tyrosine kinase 3 receptor (FLT3) and multi-targeted kinase inhibitor, in patients with acute myeloid leukemia and high-risk myelodysplastic syndrome with either wild-type or mutated FLT3. J. Clin. Oncol. 28 (28), 4339–4345. 10.1200/JCO.2010.28.9678 20733134 PMC4135183

[B28] Gao R. Kalathur R. K. R. Coto-Llerena M. Ercan C. Buechel D. Shuang S. (2021). YAP/TAZ and ATF4 drive resistance to Sorafenib in hepatocellular carcinoma by preventing ferroptosis. EMBO Mol. Med. 13 (12), e14351. 10.15252/emmm.202114351 34664408 PMC8649869

[B29] Goodman V. L. Rock E. P. Dagher R. Ramchandani R. P. Abraham S. Gobburu J. V. (2007). Approval summary: sunitinib for the treatment of imatinib refractory or intolerant gastrointestinal stromal tumors and advanced renal cell carcinoma. Clin. cancer Res. official J. Am. Assoc. Cancer Res. 13 (5), 1367–1373. 10.1158/1078-0432.CCR-06-2328 17332278

[B30] Gordon L. I. Kaplan J. B. Popat R. Burris H. A. 3rd Ferrari S. Madan S. (2020). Phase I study of TAK-659, an investigational, dual SYK/FLT3 inhibitor, in patients with B-cell lymphoma. Clin. Cancer Res. 26 (14), 3546–3556. 10.1158/1078-0432.CCR-19-3239 32327472

[B31] Grafone T. Palmisano M. Nicci C. Storti S. (2012). An overview on the role of FLT3-tyrosine kinase receptor in acute myeloid leukemia: biology and treatment. Oncol. Rev. 6 (1), e8. 10.4081/oncol.2012.e8 25992210 PMC4419636

[B32] Hage C. Hoves S. Strauss L. Bissinger S. Prinz Y. Pöschinger T. (2019). Sorafenib induces Pyroptosis in macrophages and triggers natural killer cell-mediated cytotoxicity against hepatocellular carcinoma. Hepatology 70 (4), 1280–1297. 10.1002/hep.30666 31002440

[B33] He H. Tran P. Gu H. Tedesco V. Zhang J. Lin W. (2017). Midostaurin, a novel protein kinase inhibitor for the treatment of acute myelogenous leukemia: insights from human absorption, metabolism, and excretion studies of a BDDCS II drug. Drug Metab. Dispos. 45 (5), 540–555. 10.1124/dmd.116.072744 28270565

[B34] Hosono N. Yokoyama H. Aotsuka N. Ando K. Iida H. Ishikawa T. (2021). Gilteritinib versus chemotherapy in Japanese patients with FLT3-mutated relapsed/refractory acute myeloid leukemia. Int. J. Clin. Oncol. 26 (11), 2131–2141. 10.1007/s10147-021-02006-7 34363558 PMC8522999

[B35] Huh K. Y. Hwang S. Park S. Y. Lim H. J. Jin M. Oh J. (2021). Population pharmacokinetic modelling and simulation to determine the optimal dose of nanoparticulated sorafenib to the reference sorafenib. Pharmaceutics 13 (5), 629. 10.3390/pharmaceutics13050629 33925058 PMC8145937

[B36] Jo J. C. Hong Y. S. Kim K. P. Lee J. L. Lee J. Park Y. S. (2014). A prospective multicenter phase II study of sunitinib in patients with advanced aggressive fibromatosis. Invest. New Drugs 32 (2), 369–376. 10.1007/s10637-013-0059-0 24425345

[B37] Kaplan J. B. Gordon L. I. Infante J. R. Popat R. Rambaldi A. Madan S. (2016). Updated results from a phase 1 study of TAK-659, an investigational and reversible SYK inhibitor, in patients (pts) with advanced solid tumor or lymphoma malignancies. Blood 128 (22), 624. 10.1182/blood.v128.22.624.624

[B38] Kazi J. U. Rönnstrand L. (2019). FMS-Like tyrosine kinase 3/FLT3: from basic science to clinical implications. Physiol. Rev. 99 (3), 1433–1466. 10.1152/physrev.00029.2018 31066629

[B39] Kiyoi H. Kawashima N. Ishikawa Y. (2020). FLT3 mutations in acute myeloid leukemia: therapeutic paradigm beyond inhibitor development. Cancer Sci. 111 (2), 312–322. 10.1111/cas.14274 31821677 PMC7004512

[B40] Knapper S. Burnett A. K. Littlewood T. Kell W. J. Agrawal S. Chopra R. (2006). A phase 2 trial of the FLT3 inhibitor lestaurtinib (CEP701) as first-line treatment for older patients with acute myeloid leukemia not considered fit for intensive chemotherapy. Blood 108 (10), 3262–3270. 10.1182/blood-2006-04-015560 16857985

[B41] Kolaski K. Logan L. R. Ioannidis J. P. A. (2023). Guidance to best tools and practices for systematic reviews. Br. J. Pharmacol. 12, 96. 10.1186/s13643-023-02255-9 37282770

[B42] Komrokji R. S. Seymour J. F. Roberts A. W. Wadleigh M. To L. B. Scherber R. (2015). Results of a phase 2 study of pacritinib (SB1518), a JAK2/JAK2(V617F) inhibitor, in patients with myelofibrosis. Blood 125 (17), 2649–2655. 10.1182/blood-2013-02-484832 25762180 PMC4490373

[B43] Krawczyk K. Śladowska K. Holko P. Kawalec P. (2023). Comparative safety of tyrosine kinase inhibitors in the treatment of metastatic renal cell carcinoma: a systematic review and network meta-analysis. Front. Pharmacol. 14, 1223929. 10.3389/fphar.2023.1223929 37745049 PMC10512702

[B44] Kudo M. Finn R. S. Qin S. Han K. H. Ikeda K. Cheng A. L. (2023). Overall survival and objective response in advanced unresectable hepatocellular carcinoma: a subanalysis of the REFLECT study. J. Hepatol. 78 (1), 133–141. 10.1016/j.jhep.2022.09.006 36341767

[B45] Lacy S. A. Miles D. R. Nguyen L. T. (2017). Clinical pharmacokinetics and pharmacodynamics of cabozantinib. Clin. Pharmacokinet. 56 (5), 477–491. 10.1007/s40262-016-0461-9 27734291

[B46] Lee J. H. Chen T. W. Hsu C. H. Yen Y. H. Yang J. C. Cheng A. L. (2020). A phase I study of pexidartinib, a colony-stimulating factor 1 receptor inhibitor, in Asian patients with advanced solid tumors. Invest. New Drugs 38 (1), 99–110. 10.1007/s10637-019-00745-z 30825104 PMC6985061

[B47] Lewis N. L. Lewis L. D. Eder J. P. Reddy N. J. Guo F. Pierce K. J. (2009). Phase I study of the safety, tolerability, and pharmacokinetics of oral CP-868,596, a highly specific platelet-derived growth factor receptor tyrosine kinase inhibitor in patients with advanced cancers. J. Clin. Oncol. 27 (31), 5262–5269. 10.1200/JCO.2009.21.8487 19738123 PMC2773478

[B48] Li D. Toh H. C. Merle P. Tsuchiya K. Hernandez S. Verret W. (2022). Atezolizumab plus bevacizumab versus sorafenib for unresectable hepatocellular carcinoma: results from older adults enrolled in the IMbrave150 randomized clinical trial. Liver Cancer 11 (6), 558–571. 10.1159/000525671 36589722 PMC9801180

[B49] Li J. Holmes M. Kankam M. Trone D. Mendell J. Gammon G. (2020). Effect of food on the pharmacokinetics of quizartinib. Clin. Pharmacol. Drug Dev. 9 (2), 277–286. 10.1002/cpdd.770 31916418 PMC7027461

[B50] Lin S. M. Lu S. N. Chen P. T. Jeng L. B. Chen S. C. Hu C. T. (2017). HATT: a phase IV, single-arm, open-label study of sorafenib in Taiwanese patients with advanced hepatocellular carcinoma. Hepatol. Int. 11 (2), 199–208. 10.1007/s12072-016-9774-x 27909950 PMC5362674

[B51] Lv K. Ren J. G. Han X. Gui J. Gong C. Tong W. (2021). Depalmitoylation rewires FLT3-ITD signaling and exacerbates leukemia progression. Blood 138 (22), 2244–2255. 10.1182/blood.2021011582 34111291 PMC8832469

[B52] Marshall J. L. Kindler H. Deeken J. Bhargava P. Vogelzang N. J. Rizvi N. (2005). Phase I trial of orally administered CEP-701, a novel neurotrophin receptor-linked tyrosine kinase inhibitor. Invest. New Drugs 23 (1), 31–37. 10.1023/B:DRUG.0000047103.64335.b0 15528978

[B53] Matulonis U. A. Sill M. W. Makker V. Mutch D. G. Carlson J. W. Darus C. J. (2019). A randomized phase II study of cabozantinib versus weekly paclitaxel in the treatment of persistent or recurrent epithelial ovarian, fallopian tube or primary peritoneal cancer: an NRG Oncology/Gynecologic Oncology Group study. Gynecol. Oncol. 152 (3), 548–553. 10.1016/j.ygyno.2018.12.008 30587441 PMC6542283

[B54] Mesa R. A. Vannucchi A. M. Mead A. Egyed M. Szoke A. Suvorov A. (2017). Pacritinib versus best available therapy for the treatment of myelofibrosis irrespective of baseline cytopenias (PERSIST-1): an international, randomised, phase 3 trial. Lancet Haematol. 4 (5), e225–e236. 10.1016/S2352-3026(17)30027-3 28336242 PMC8209752

[B55] Nakaigawa N. Tomita Y. Tamada S. Tatsugami K. Osawa T. Oya M. (2023). Final efficacy and safety results and biomarker analysis of a phase 2 study of cabozantinib in Japanese patients with advanced renal cell carcinoma. Int. J. Clin. Oncol. 28 (3), 416–426. 10.1007/s10147-022-02283-w 36595123 PMC9988754

[B56] NCT00379080 (2017). Clinicaltrials. Available at: https://clinicaltrials.gov/study/NCT00379080?cond=NCT00379080&rank=1&tab=results. Accessed on April 7, 2017.

[B57] Nguyen L. Benrimoh N. Xie Y. Offman E. Lacy S. (2016). Pharmacokinetics of cabozantinib tablet and capsule formulations in healthy adults. Anticancer Drugs 27 (7), 669–678. 10.1097/CAD.0000000000000366 27139820

[B58] Numan Y. Abdel Rahman Z. Grenet J. Boisclair S. Bewersdorf J. P. Collins C. (2022). Gilteritinib clinical activity in relapsed/refractory FLT3 mutated acute myeloid leukemia previously treated with FLT3 inhibitors. Am. J. Hematol. 97 (3), 322–328. 10.1002/ajh.26447 34981560

[B59] O’Farrell A. M. Foran J. M. Fiedler W. Serve H. Paquette R. L. Cooper M. A. (2003). An innovative phase I clinical study demonstrates inhibition of FLT3 phosphorylation by SU11248 in acute myeloid leukemia patients. Clin. Cancer Res. 9 (15), 5465–5476. 14654525

[B60] Perl A. E. Altman J. K. Cortes J. Smith C. Litzow M. Baer M. R. (2017). Selective inhibition of FLT3 by gilteritinib in relapsed or refractory acute myeloid leukaemia: a multicentre, first-in-human, open-label, phase 1-2 study. Lancet Oncol. 18 (8), 1061–1075. 10.1016/S1470-2045(17)30416-3 28645776 PMC5572576

[B61] Perl A. E. Martinelli G. Cortes J. E. Neubauer A. Berman E. Paolini S. (2019). Gilteritinib or chemotherapy for relapsed or refractory *FLT3*-mutated AML. N. Engl. J. Med. 381 (18), 1728–1740. 10.1056/NEJMoa1902688 31665578

[B62] Pratz K. W. Kaplan J. Levy M. Bixby D. Burke P. W. Erba H. (2023). A phase Ib trial of mivavotinib (TAK-659), a dual SYK/FLT3 inhibitor, in patients with relapsed/refractory acute myeloid leukemia. Haematologica 108 (3), 705–716. 10.3324/haematol.2022.281216 36226495 PMC9973464

[B63] Procopio G. Claps M. Pircher C. Porcu L. Sepe P. Guadalupi V. (2023). A multicenter phase 2 single arm study of cabozantinib in patients with advanced or unresectable renal cell carcinoma pre-treated with one immune-checkpoint inhibitor: the BREAKPOINT trial (Meet-Uro trial 03). Tumori 109 (1), 129–137. 10.1177/03008916221138881 36447337 PMC9896529

[B64] Propper D. J. McDonald A. C. Man A. Thavasu P. Balkwill F. Braybrooke J. P. (2001). Phase I and pharmacokinetic study of PKC412, an inhibitor of protein kinase C. J. Clin. Oncol. 19 (5), 1485–1492. 10.1200/JCO.2001.19.5.1485 11230495

[B65] Schlenk R. F. Dohner K. Krauter J. Fröhling S. Corbacioglu A. Bullinger L. (2008). Mutations and treatment outcome in cytogenetically normal acute myeloid leukemia. N. Engl. J. Med. 358, 1909–1918. 10.1056/NEJMoa074306 18450602

[B66] Semrad T. J. Eddings C. Pan C. X. Lau D. H. Gandara D. Beckett L. (2012). Feasibility study of intra-patient sorafenib dose-escalation or re-escalation in patients with previously treated advanced solid tumors. Invest. New Drugs 30 (5), 2001–2007. 10.1007/s10637-011-9761-y 22015991 PMC4131984

[B67] Senapati J. Kadia T. M. (2022). Which FLT3 inhibitor for treatment of AML? Curr. Treat. Options Oncol. 23 (3), 359–380. 10.1007/s11864-022-00952-6 35258791

[B68] Shepard D. R. Cooney M. M. Elson P. Bukowski R. M. Dreicer R. Rini B. I. (2012). A phase II study of tandutinib (MLN518), a selective inhibitor of type III tyrosine receptor kinases, in patients with metastatic renal cell carcinoma. Invest. New Drugs 30 (1), 364–367. 10.1007/s10637-010-9516-1 20711630

[B69] Smith B. D. Levis M. Beran M. Giles F. Kantarjian H. Berg K. (2004). Single-agent CEP-701, a novel FLT3 inhibitor, shows biologic and clinical activity in patients with relapsed or refractory acute myeloid leukemia. Blood 103 (10), 3669–3676. 10.1182/blood-2003-11-3775 14726387

[B70] Smith C. C. Levis M. J. Frankfurt O. Pagel J. M. Roboz G. J. Stone R. M. (2020). A phase 1/2 study of the oral FLT3 inhibitor pexidartinib in relapsed/refractory FLT3-ITD-mutant acute myeloid leukemia. Blood Adv. 4 (8), 1711–1721. 10.1182/bloodadvances.2020001449 32330242 PMC7189289

[B71] Sun W. Li S. C. Xu L. Zhong W. Wang Z. G. Pan C. Z. (2020). High FLT3 levels may predict sorafenib benefit in hepatocellular carcinoma. Clin. Cancer Res. 26 (16), 4302–4312. 10.1158/1078-0432.CCR-19-1858 32332018

[B72] Takahashi S. (2011). Downstream molecular pathways of FLT3 in the pathogenesis of acute myeloid leukemia: biology and therapeutic implications. J. Hematol. Oncol. 4, 13. 10.1186/1756-8722-4-13 21453545 PMC3076284

[B73] Tap W. D. Gelderblom H. Palmerini E. Desai J. Bauer S. Blay J. Y. (2019). Pexidartinib versus placebo for advanced tenosynovial giant cell tumour (ENLIVEN): a randomised phase 3 trial. Lancet 394 (10197), 478–487. 10.1016/S0140-6736(19)30764-0 31229240 PMC6860022

[B74] Usuki K. Handa H. Choi I. Yamauchi T. Iida H. Hata T. (2019). Safety and pharmacokinetics of quizartinib in Japanese patients with relapsed or refractory acute myeloid leukemia in a phase 1 study. Int. J. Hematol. 110 (6), 654–664. 10.1007/s12185-019-02709-8 31359361

[B75] Usuki K. Sakura T. Kobayashi Y. Miyamoto T. Iida H. Morita S. (2018). Clinical profile of gilteritinib in Japanese patients with relapsed/refractory acute myeloid leukemia: an open-label phase 1 study. Cancer Sci. 109 (10), 3235–3244. 10.1111/cas.13749 30039554 PMC6172068

[B76] Verstovsek S. Odenike O. Singer J. W. Granston T. Al-Fayoumi S. Deeg H. J. (2016). Phase 1/2 study of pacritinib, a next generation JAK2/FLT3 inhibitor, in myelofibrosis or other myeloid malignancies. J. Hematol. Oncol. 9 (1), 137. 10.1186/s13045-016-0367-x 27931243 PMC5146859

[B77] Wang Y. Yin O. Q. Graf P. Kisicki J. C. Schran H. (2008). Dose- and time-dependent pharmacokinetics of midostaurin in patients with diabetes mellitus. J. Clin. Pharmacol. 48 (6), 763–775. 10.1177/0091270008318006 18508951

[B78] Wu D. Jia B. Jia M. Zhao H. Zhao H. Zhou J. (2023). Comparative efficacy and safety of systemic therapy for advanced hepatocellular carcinoma: a systematic review and network meta-analysis. Front. Oncol. 13, 1274754. 10.3389/fonc.2023.1274754 38125936 PMC10730675

[B79] Xu R. H. Shen L. Wang K. M. Wu G. Shi C. M. Ding K. F. (2017). Famitinib versus placebo in the treatment of refractory metastatic colorectal cancer: a multicenter, randomized, double-blinded, placebo-controlled, phase II clinical trial. Chin. J. Cancer 36 (1), 97. 10.1186/s40880-017-0263-y 29273089 PMC5741870

[B80] Yazdi M. H. Faramarzi M. A. Nikfar S. Abdollahi M. (2017). Comparative safety and efficacy of tyrosine kinase inhibitors (TKIs) in the treatment setting of different types of leukemia, and different types of adenocarcinoma. Biomed. Pharmacother. 95, 1556–1564. 10.1016/j.biopha.2017.09.088 28950655

[B81] Younes A. Romaguera J. Fanale M. McLaughlin P. Hagemeister F. Copeland A. (2012). Phase I study of a novel oral Janus kinase 2 inhibitor, SB1518, in patients with relapsed lymphoma: evidence of clinical and biologic activity in multiple lymphoma subtypes. J. Clin. Oncol. 30 (33), 4161–4167. 10.1200/JCO.2012.42.5223 22965964 PMC5950499

[B82] Yuan S. Wei C. Liu G. Zhang L. Li J. Li L. (2022). Sorafenib attenuates liver fibrosis by triggering hepatic stellate cell ferroptosis via HIF-1α/SLC7A11 pathway. Cell Prolif. 55 (1), e13158. 10.1111/cpr.13158 34811833 PMC8780895

[B83] Zahir H. Yin O. Hsu C. Wagner A. J. Jiang J. Wang X. (2023). Dosing recommendation based on the effects of different meal types on pexidartinib pharmacokinetics in healthy subjects: implementation of model-informed drug development strategy. Clin. Pharmacol. Drug Dev. 12 (5), 475–483. 10.1002/cpdd.1240 36942508

[B84] Zeng X. Zhang Y. Kwong J. S. Zhang C. Li S. Sun F. (2015). The methodological quality assessment tools for preclinical and clinical studies, systematic review and meta-analysis, and clinical practice guideline: a systematic review. J. Evid. Based Med. 8 (1), 2–10. 10.1111/jebm.12141 25594108

[B85] Zhang W. Zhou A. P. Qin Q. Chang C. X. Jiang H. Y. Ma J. H. (2013). Famitinib in metastatic renal cell carcinoma: a single center study. Chin. Med. J. Engl. 126 (22), 4277–4281. 10.3760/cma.j.issn.0366-6999.20131757 24238512

[B86] Zhao J. C. Agarwal S. Ahmad H. Amin K. Bewersdorf J. P. Zeidan A. M. (2022). A review of FLT3 inhibitors in acute myeloid leukemia. Blood Rev. 52, 100905. 10.1016/j.blre.2021.100905 34774343 PMC9846716

[B87] Zhou A. Zhang W. Chang C. Chen X. Zhong D. Qin Q. (2013). Phase I study of the safety, pharmacokinetics and antitumor activity of famitinib. Cancer Chemother. Pharmacol. 72 (5), 1043–1053. 10.1007/s00280-013-2282-y 24043137

[B88] Zou H. Pan T. Gao Y. Chen R. Li S. Guo J. (2022). Pan-cancer assessment of mutational landscape in intrinsically disordered hotspots reveals potential driver genes. Nucleic Acids Res. 50 (9), e49. 10.1093/nar/gkac028 35061901 PMC9122534

