# Supplementary figures and images for: Efficacy and safety of FLT3 inhibitors in monotherapy of hematological and solid malignancies: a systemic analysis of clinical trials
Source: Front Pharmacol. 2024 May 17;15:1294668. doi: 10.3389/fphar.2024.1294668 (PMC11140126; doi:10.3389/fphar.2024.1294668)

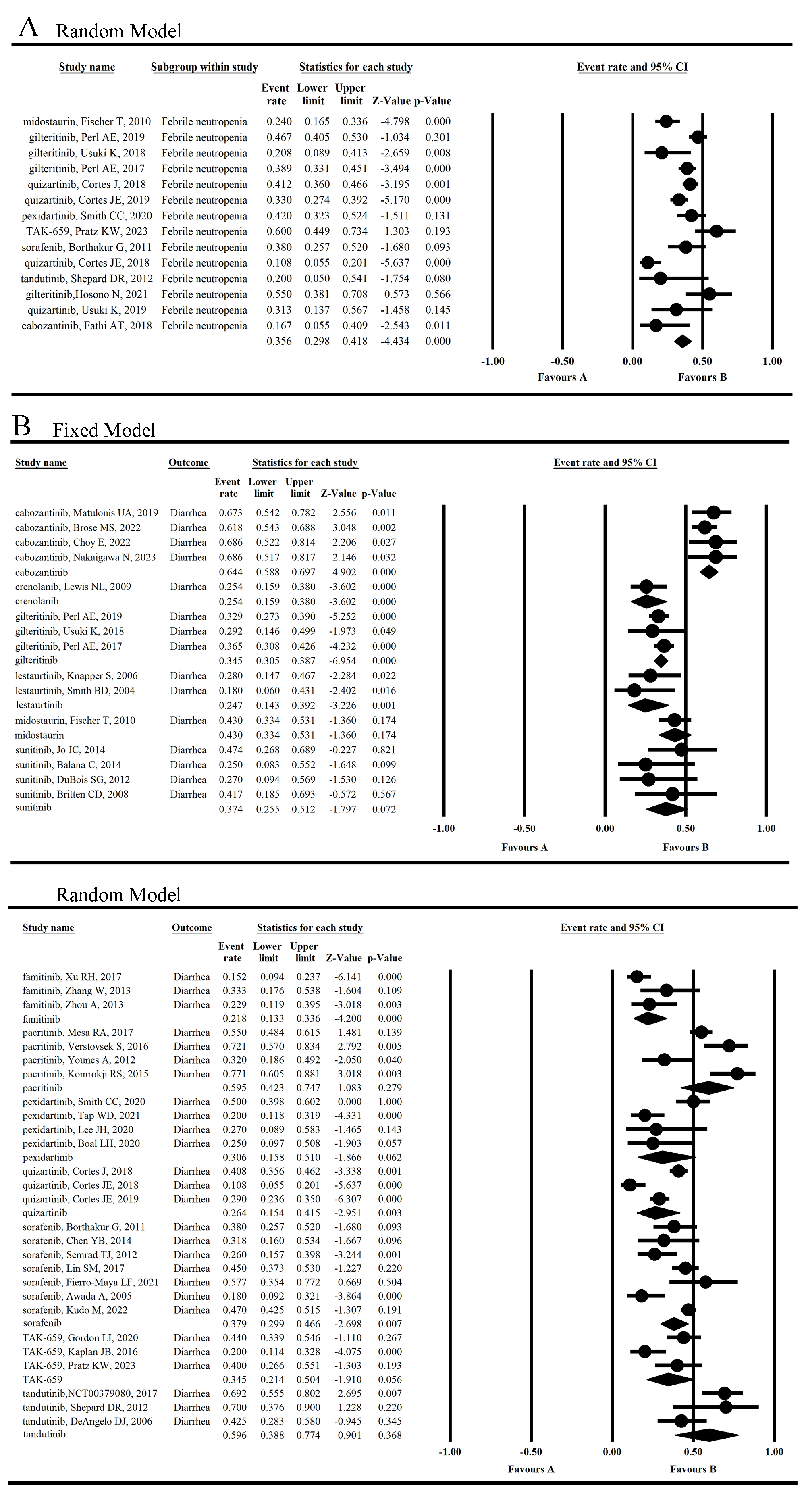

Supplement: Supplementary file 1 [file Image6.TIF]

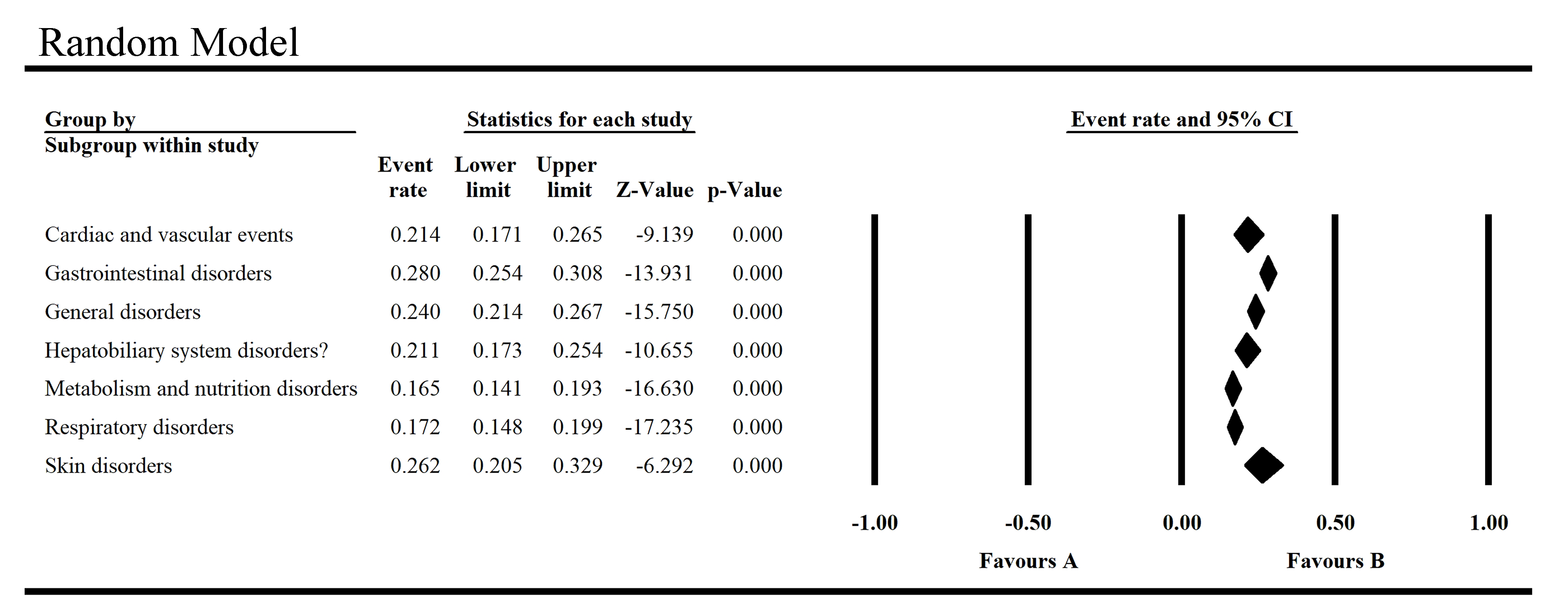

Supplement: Supplementary file 3 [file Image3.TIF]

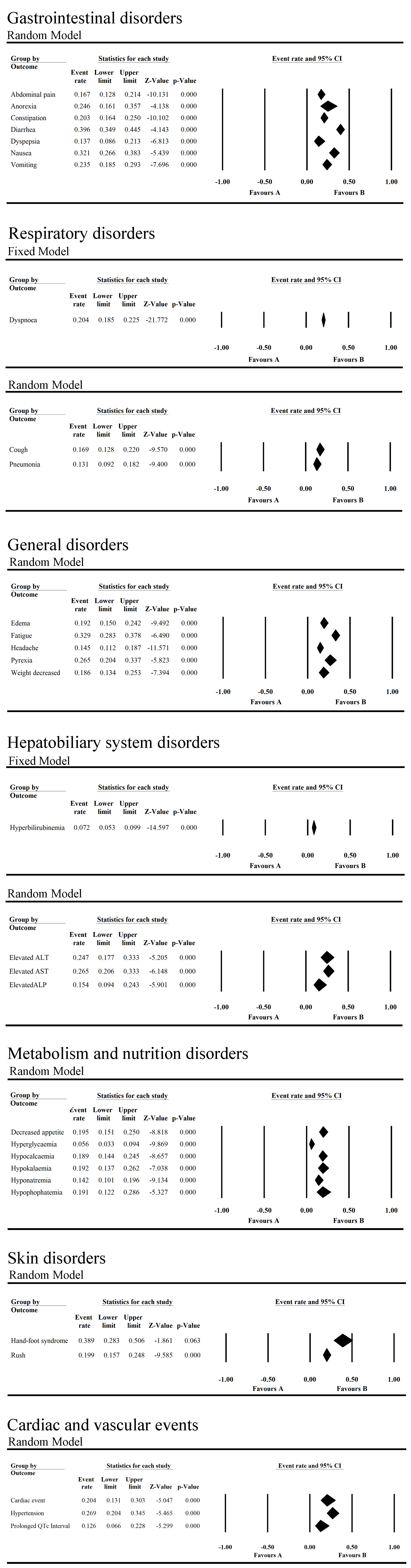

Supplement: Supplementary file 4 [file Image4.TIF]

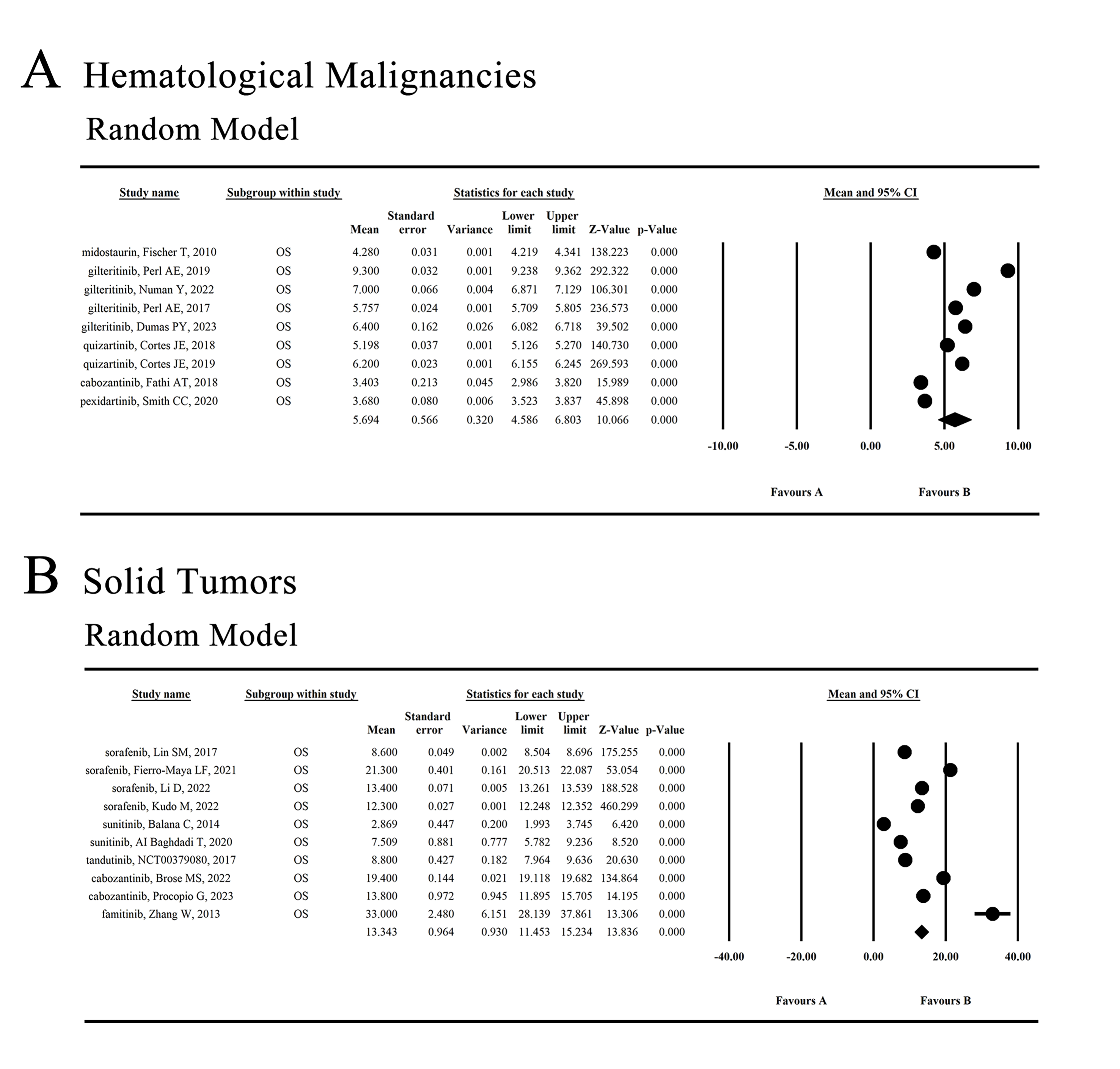

Supplement: Supplementary file 5 [file Image9.TIF]

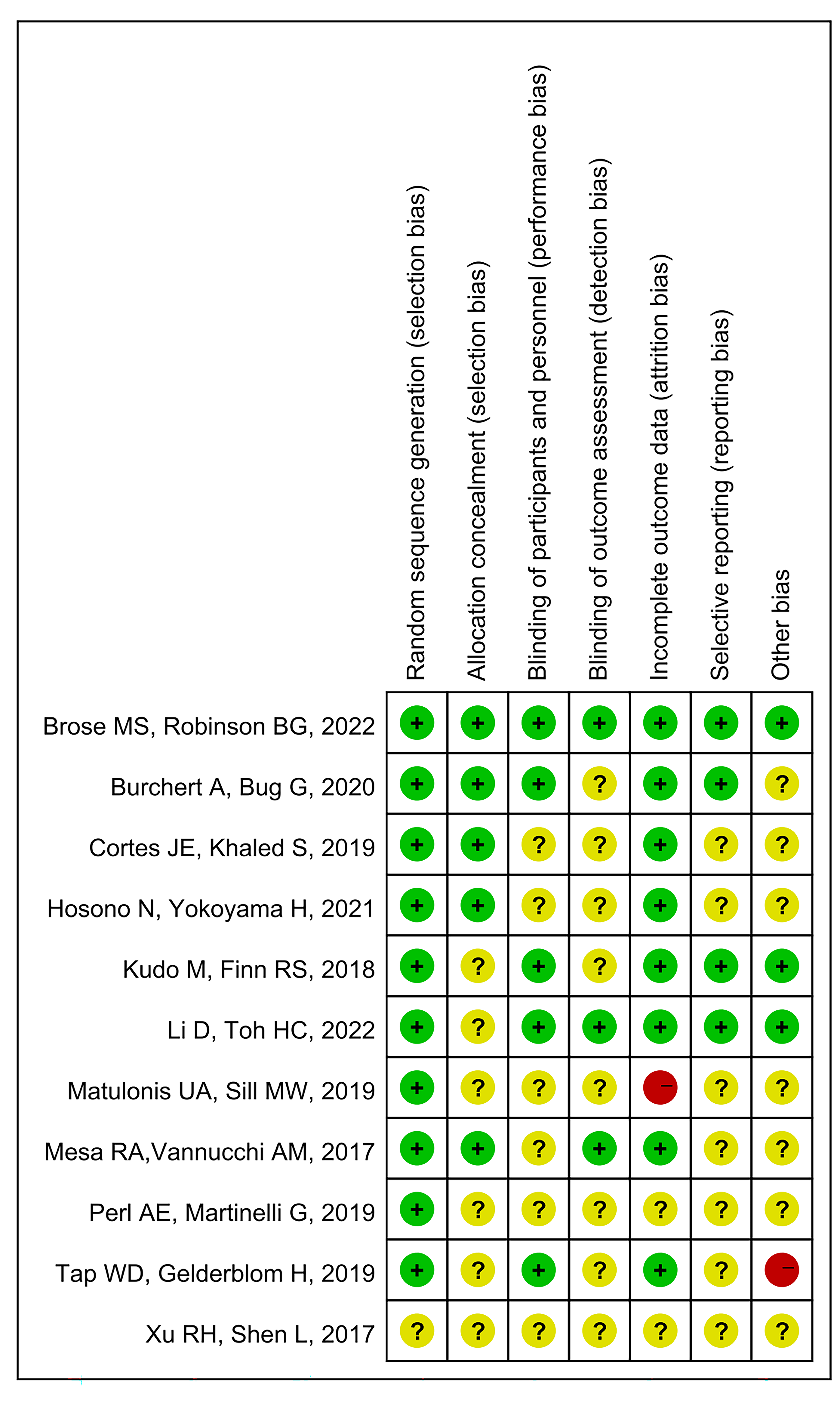

Supplement: Supplementary file 6 [file Image2.TIF]

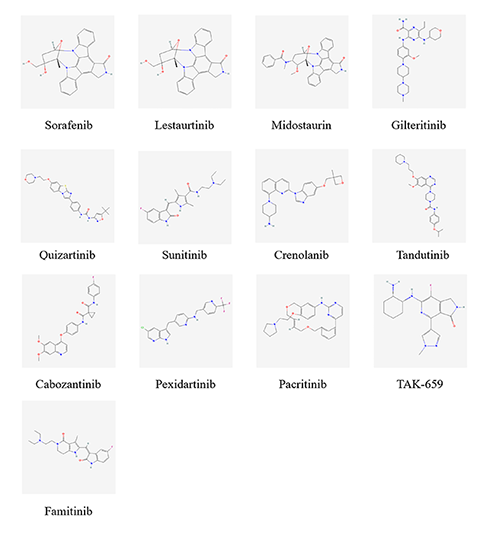

Supplement: Supplementary file 7 [file Image1.TIF]

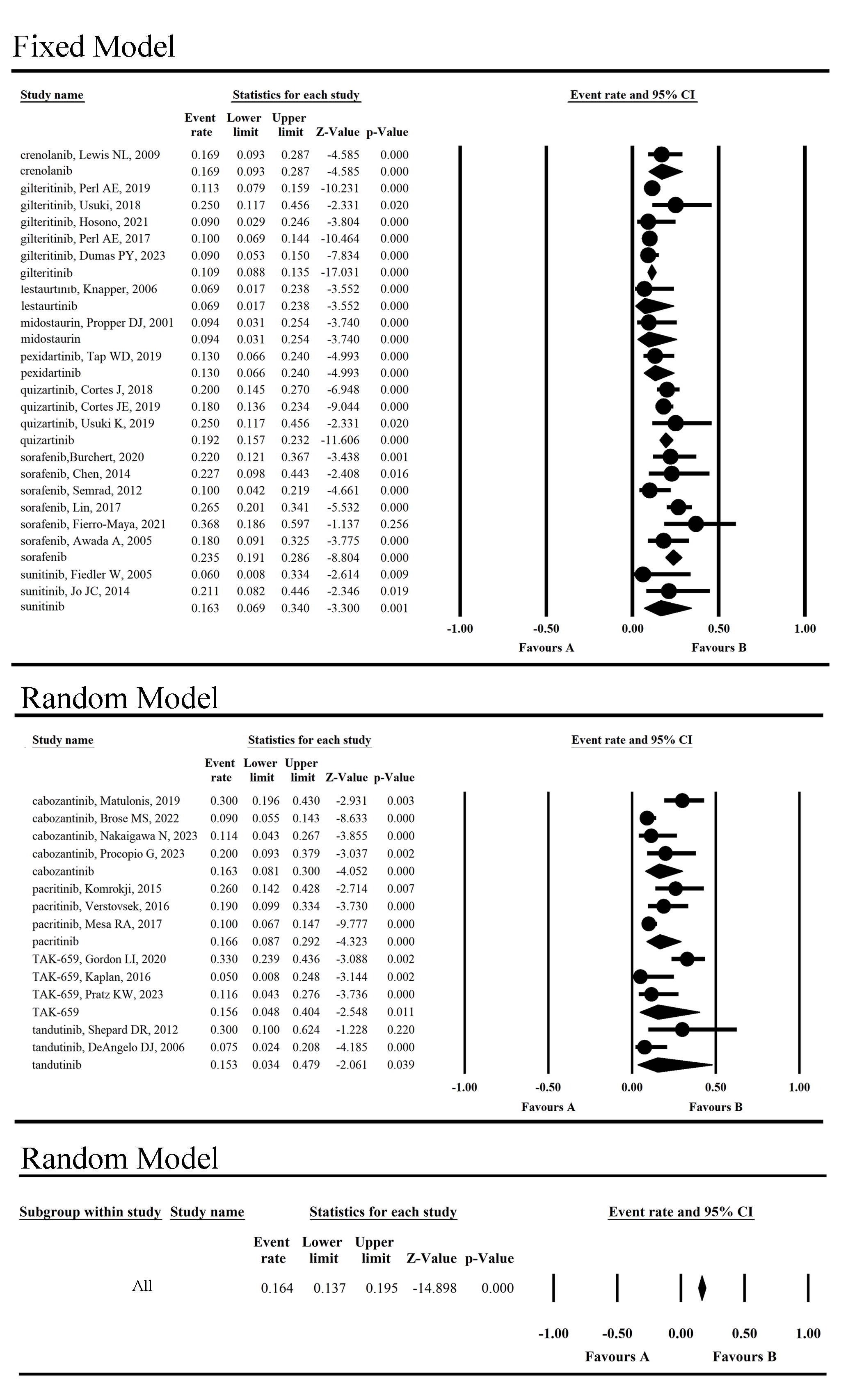

Supplement: Supplementary file 8 [file Image7.TIF]

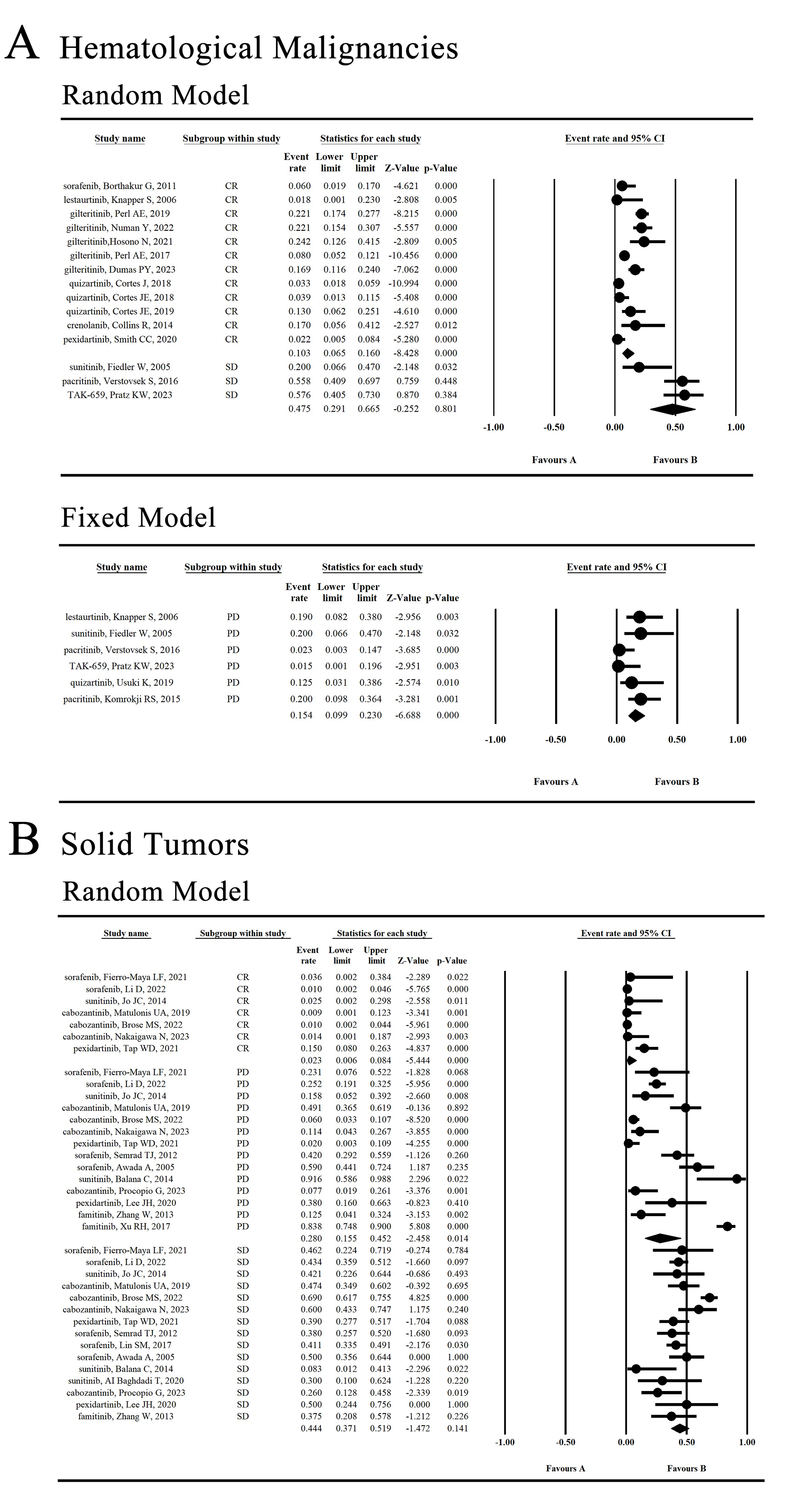

Supplement: Supplementary file 9 [file Image8.TIF]

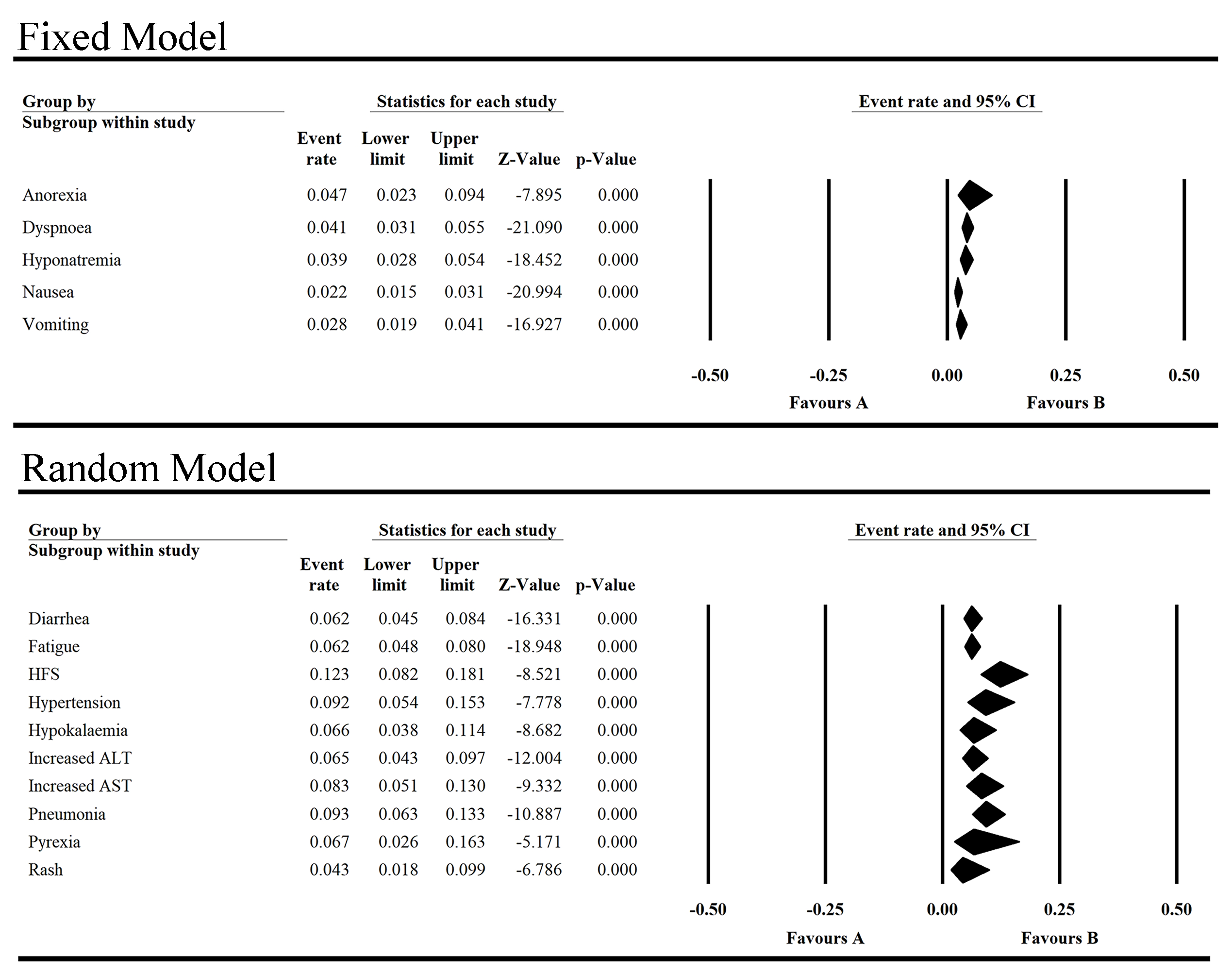

Supplement: Supplementary file 10 [file Image5.TIF]
