# Supplementary material for: Efficacy and safety of FLT3 inhibitors in monotherapy of hematological and solid malignancies: a systemic analysis of clinical trials
Source: Front Pharmacol. 2024 May 17;15:1294668. doi: 10.3389/fphar.2024.1294668 (PMC11140126; doi:10.3389/fphar.2024.1294668)
Supplement: Supplementary file 2 [file Table1.docx]

**Supplementary Table 1 |** The research assessment scores of included articles according to MINORS

| **Author**  **Publication time** | **A clearly stated aim** | **Inclusion of consecutive patients** | **Prospective collection of data** | **Endpoints appropriate to the aim of the study** | **Unbiased assessment of the study endpoint** | **Follow-up period appropriate to the aim of the study** | **Loss to follow up less than 5%** | **Prospective calculation of the study size** | **Total** |
| --- | --- | --- | --- | --- | --- | --- | --- | --- | --- |
| Borthakur G, Kantarjian H, 2011 | ★★ | ★★ | ★★ | ★★ | ★★ | ★★ | ★★ | ★ | 15 |
| Chen YB, Li S, 2014 | ★★ | ★★ | ★ | ★ | ★★ | ★★ | ★ | ★ | 12 |
| Semrad TJ, Eddings C, 2012 | ★★ | ★ | ★ | ★★ | ★★ | ★ | ★★ | ★ | 12 |
| Lin SM, Lu SN, 2017 | ★★ | ★★ | ★★ | ★★ | ★★ | ★★ | ★★ | ★★ | 16 |
| Fierro-Maya LF, González GG, 2021 | ★★ | ★★ | ★★ | ★★ | ★★ | ★★ | ★★ | ★ | 15 |
| Huh KY, Hwang S, 2021 | ★★ | ★★ | ★★ | ★★ | ★★ | ★ | ★★ | ★★ | 15 |
| Awada A, Hendlisz A, 2005 | ★★ | ★★ | ★★ | ★★ | ★★ | ★★ | ★★ | ★★ | 16 |
| Knapper S, Burnett AK, 2006 | ★★ | ★ | ★ | ★★ | ★★ | ★★ | ★★ | ★ | 14 |
| Smith BD, Levis M, 2004 | ★★ | ★★ | ★★ | ★★ | ★★ | ★★ | ★★ | ★★ | 16 |
| Marshall JL, Kindler H, 2005 | ★★ | ★★ | ★ | ★★ | ★★ | ★★ | ★★ | ★ | 14 |
| Fischer T, Stone RM, 2010 | ★★ | ★★ | ★ | ★★ | ★★ | ★★ | ★★ | ★★ | 15 |
| Propper DJ, McDonald AC, 2001 | ★★ | ★★ | ★★ | ★★ | ★★ | ★ | ★★ | ★★ | 15 |
| He H, Tran P, 2017 | ★★ | ★★ | ★★ | ★★ | ★★ | ★ | ★★ | ★★ | 15 |
| Numan Y, Abdel Rahman Z, 2022 | ★★ | ★★ | ★ | ★★ | ★★ | ★★ | ★★ | ★★ | 15 |
| Usuki K, Sakura T, 2018 | ★★ | ★★ | ★★ | ★★ | ★★ | ★★ | ★★ | ★★ | 16 |
| Perl AE, Altman JK, 2017 | ★★ | ★★ | ★★ | ★★ | ★★ | ★★ | ★★ | ★★ | 16 |
| Dumas PY, Raffoux E, 2023 | ★★ | ★★ | ★★ | ★★ | ★★ | ★★ | ★★ | ★★ | 16 |
| Cortes J, Perl AE, 2018 | ★★ | ★★ | ★★ | ★★ | ★★ | ★ | ★★ | ★★ | 15 |
| Cortes JE, Tallman MS, 2018 | ★★ | ★★ | ★★ | ★★ | ★★ | ★★ | ★★ | ★★ | 16 |
| Usuki K, Handa H, 2019 | ★★ | ★★ | ★ | ★★ | ★★ | ★★ | ★★ | ★ | 14 |
| Li J, Holmes M, 2020 | ★★ | ★★ | ★★ | ★★ | ★★ | ★ | ★ | ★★ | 14 |
| Fiedler W, Serve H, 2005 | ★★ | ★★ | ★★ | ★★ | ★★ | ★★ | ★★ | ★★ | 16 |
| Jo JC, Hong YS, 2014 | ★★ | ★★ | ★★ | ★★ | ★★ | ★★ | ★★ | ★ | 15 |
| Balaña C, Balaña C, 2014 | ★★ | ★★ | ★ | ★ | ★★ | ★★ | ★★ | ★★ | 14 |
| Al Baghdadi T, Garrett-Mayer E, 2020 | ★★ | ★ | ★ | ★★ | ★★ | ★ | ★★ | ★ | 12 |
| DuBois SG, Shusterman S, 2012 | ★★ | ★★ | ★ | ★★ | ★★ | ★ | ★★ | ★★ | 14 |
| Britten CD, Kabbinavar F, 2008 | ★★ | ★★ | ★★ | ★ | ★★ | ★ | ★★ | ★★ | 14 |
| O'Farrell AM, Foran JM, 2003 | ★★ | ★★ | ★★ | ★★ | ★★ | ★★ | ★★ | ★★ | 16 |
| Faivre S, Delbaldo C, 2006 | ★★ | ★★ | ★★ | ★★ | ★★ | ★★ | ★★ | ★ | 15 |
| Cortes JE, Kantarjian HM, 2016 | ★★ | ★★ | ★ | ★★ | ★★ | ★★ | ★ | ★★ | 14 |
| Collins R, Kantarjian HM, 2014 | ★★ | ★★ | ★ | ★★ | ★★ | ★ | ★ | ★ | 12 |
| Lewis NL, Lewis LD, 2009 | ★★ | ★★ | ★★ | ★★ | ★★ | ★ | ★★ | ★★ | 15 |
| NCT00379080, Batchelor TT, 2017 | ★★ | ★★ | ★★ | ★★ | ★ | ★★ | ★★ | ★★ | 15 |
| Shepard DR, Cooney MM, 2012 | ★★ | ★ | ★ | ★★ | ★ | ★ | ★★ | ★ | 11 |
| DeAngelo DJ, Stone RM, 2006 | ★★ | ★★ | ★★ | ★★ | ★ | ★★ | ★★ | ★ | 14 |
| Fathi AT, Blonquist TM, 2018 | ★★ | ★★ | ★★ | ★ | ★★ | ★ | ★★ | ★ | 13 |
| Nguyen L, Benrimoh N, 2016 | ★★ | ★★ | ★★ | ★★ | ★★ | ★ | ★★ | ★ | 14 |
| Choy E, Cote GM, 2022 | ★★ | ★★ | ★★ | ★★ | ★ | ★★ | ★★ | ★ | 14 |
| Nakaigawa N, Tomita Y, 2023 | ★★ | ★★ | ★★ | ★★ | ★★ | ★★ | ★★ | ★ | 15 |
| Procopio G, Claps M, 2023 | ★★ | ★★ | ★★ | ★★ | ★★ | ★★ | ★★ | ★ | 15 |
| Smith CC, Levis MJ,2020 | ★★ | ★★ | ★ | ★ | ★★ | ★ | ★★ | ★ | 12 |
| Lee JH, Chen TW, 2020 | ★★ | ★★ | ★ | ★★ | ★★ | ★★ | ★★ | ★★ | 15 |
| Boal LH, Glod J, 2020 | ★★ | ★★ | ★ | ★★ | ★★ | ★★ | ★★ | ★ | 14 |
| Verstovsek S, Odenike O, 2016 | ★★ | ★★ | ★ | ★★ | ★★ | ★ | ★★ | ★★ | 14 |
| Younes A, Romaguera J, 2012 | ★★ | ★★ | ★★ | ★★ | ★★ | ★★ | ★★ | ★★ | 16 |
| Komrokji RS, Seymour JF, 2015 | ★★ | ★★ | ★★ | ★★ | ★★ | ★ | ★★ | ★★ | 15 |
| Gordon LI, Kaplan JB, 2020 | ★★ | ★★ | ★ | ★★ | ★★ | ★★ | ★★ | ★★ | 15 |
| Kaplan JB, Gordon LI, 2016 | ★★ | ★★ | ★ | ★ | ★★ | ★★ | ★★ | ★ | 13 |
| Pratz KW, Kaplan J, 2023 | ★★ | ★★ | ★★ | ★★ | ★ | ★★ | ★ | ★ | 14 |
| Zhang W, Zhou AP, 2013 | ★★ | ★★ | ★★ | ★★ | ★★ | ★★ | ★★ | ★★ | 16 |
| Zhou A, Zhang W, 2013 | ★★ | ★★ | ★ | ★★ | ★★ | ★★ | ★★ | ★★ | 15 |

**Supplementary Table 2** **| DLT and Emergency adverse reactions causing the discontinuation in FLT3 inhibitor monotherapy.**

|  | DLT | Emergency adverse events causing discontinuation |
| --- | --- | --- |
| sorafenib | weight loss,^15^ tongue/facial swelling,^15^ nausea,^15^ diarrhea,^15,20^ fatigue,^20^ anorexia,^20^ vomiting,^20^ nausea,^20^ pain,^20^ abdominal cramping,^20^ retrosternal pain,^20^ oedema of uvula^20^ | myocardial infarction,^18^ and other AEs which led to discontinuation of 22% of the patients^14,16,17,20^ (no specific AEs were mentioned) |
| lestaurtinib | nausea,^25^ anemia,^25^ anorexia,^25^ dyspepsia,^25^ asthenia,^25^ dehydration^25^ | fatigue^23^ |
| midostaurin | lethargy,^27^ fatigue,^27^ nausea,^27^ vomiting^27^ | nausea,^27^ vomiting,^27^ cerebral vascular (thrombotic) accident ^27^ |
| gilteritinib | tumor lysis syndrome,^31^ syncope,^31^ elevated amylase,^31^ elevated blood creatine phosphokinase,^31^ elevated blood lactate dehydrogenase^31^ | elevated aspartate aminotransferase,^29^ elevated alanine aminotransferase,^29^ pneumonia,^29,31,32^ subdural hematoma,^31^ elevated blood creatine phosphokinase,^31^ elevated lactate dehydrogenase,^31^delirium,^31^ hyperglycemia,^32^ intestinal ischemia,^32^ sepsis,^32,33^ back pain,^32^ depressed consciousness,^32^ cholecystitis^32^ |
| quizartinib | QT interval prolongation^37^ | pericardial effusion,^36^ pericarditis,^36^ diarrhea,^36^ neutropenic sepsis,^36^ pleural effusion,^36^ QT prolongation,^35,37^ pneumonia,^37^ intracranial haemorrhage,^37^ graft-versus-host disease,^37^ sepsis or septic shock,^37^ bronchopulmonary aspergillosis^38^ |
| sunitinib | dizziness with back pain,^44^ hand–foot syndrome,^44^ hypoxia,^44^ proteinuria,^44^ alkaline phosphatase,^44^ asthenia,^47^ hypertension,^47^ tumor necrosis,^47^ edema,^47^ peripheral thrombocytopenia,^47^ and other AEs (no specific AEs mentioned)^46^ | fatigue,^40^ hypertension,^40^ and other AEs (no specific AEs mentioned)^41^ |
| crenolanib | hematuria,^50^ increased glutamyltransferase or ALT,^50^ insomnia,^50^ nausea,^50^ vomiting^50^ | nausea,^50^ vomiting,^50^ reversible elevations in hepatic enzymes^50^ |
| tandutinib | generalized muscular weakness,^53^ diarrhea,^53^ fatigue^53^ | weakness,^53^ and other AEs (no specific AEs mentioned)^52^ |
| cabozantinib | pancreatitis,^54^ transaminitis^54^ | hyponatriemia,^60^ and other AEs (no specific AEs mentioned)^57,59,60^ |
| pexidartinib | eight AEs (no specific AEs mentioned)^63^ | Hepatic AEs^62^ |
| pacritinib | anemia,^65^ thrombocytopenia,^65^ QT prolongation,^66^ diarrhea,^66^ nausea,^66^ vomiting,^66^ dizziness,^66^ gait disturbance,^66^ performance status decreased,^66^ vision blurred^66^ | gastrointestinal adverse events,^65^ diarrhea,^65,66^ QTc prolongation,^66^ fatigue,^66^ increased transaminases,^66^ anemia,^66^ hypersensitivity,^68^ pruritus,^68^ thrombocytopenia,^68^ hyperbilirubinemia,^68^ subdural hematoma,^68^ nausea^68^ |
| TAK-659 | increased aspartate aminotransferase,^69-72^ stomatitis,^69^ generalized edema,^69-71^ increased lipase,^69-72^ hypophosphatemia,^69^ mucositis,^70,71^ gastrointestinal bleeding^72^ | AEs (no specific AEs mentioned),^69^ sepsis,^70^ pneumonitis,^70^ pneumonia,^71^ gastrointestinal- or central nervous system-related bleeding^72^ |
| famitinib | hypertension,^74^ thrombocytopenia,^74^ hand-foot skin reaction,^74^ diarrhea^74^ | - |

**Supplementary Table 3 |** **IC50 of FLT3 inhibitors.**

| FLT3 inhibitors | Tumor type | DOI | Cell lines | IC50 (24H) | IC50 (48H) | IC50 (72H) |
| --- | --- | --- | --- | --- | --- | --- |
| sorafenib | hepatocellular carcinoma | 10.1186/s13046-022-02567-z | Huh7  SMMC-7721 | 3.68 ± 0.75μM  7.68±0.82μM | NA | NA |
| lestaurtinib | hepatocellular carcinoma | 10.3389/fcell.2022.837428 | Huh7 | >4 μM | 0.67 ± 0.17μM | 0.25 ± 0.13 μM |
| midostaurin | Colon Cancer | 10.3390/cancers14194847 | HCT116  SW480 | 3.6μM  8.8μM | NA | NA |
| gilteritinib | Acute myeloid leukemia | 10.18632/oncotarget.27489 | MOLM-13  MOLM-14 | NA | NA | 10.00 nM  7.41 nM |
| quizartinib | Acute myeloid leukemia | 10.18632/oncotarget.27489 | MOLM-13  MOLM-14 | NA | NA | 0.89 nM  0.73 nM |
| sunitinib | Acute myeloid leukemia | 10.1007/s00277-012-1627-7 | HL-60  KG-1 | NA | 4.5μM  3.9μM | NA |
| crenolanib | Acute myeloid leukemia | 10.18632/oncotarget.27489 | MOLM-13  MOLM-14 | NA | NA | 3.86 nM  3.19 nM |
| tandutinib | Leukemia | 10.1016/s1535-6108(02)00070-3 | MOLM-13  MOLM-14  KG-1  RS4;11  THP-1 | NA | NA | 0.01μM  0.01μM  25μM  12Μm  13μM |
| cabozantinib | Acute myeloid leukemia | 10.1016/j.canlet.2016.04.004 | MOLM-13  THP-1 | NA | NA | 2.0±0.8μM  20.3±2.1μM |
| pexidartinib | Anaplastic Thyroid Cancer | 10.3390/cancers15010172 | CAL-62  BHT101 | 6.413 μM  8.482 μM | 4.116 μM  5.342 μM | 2.925 μM  3.511 μM |
| pacritinib | Acute myeloid leukemia | 10.1038/leu.2011.148 | MV4-11  MOLM-13 | NA | 47nM  67nM | NA |
| famitinib | Non-small cell lung cancer | 10.1111/1759-7714.13902 | NCI‐H292  PC‐9  NCI‐H1975 | NA | NA | 2956.5± 486.5nM  3590.0± 241.4nM  3816.7± 537.3nM |
| TAK-659 | Hematopoietic malignancies | 10.1200/JCO.2016.34.15_SUPPL.E14091 | hematopoietic-derived cell lines | 11 to 775 nM | NA | NA |

Note: NA, not available.

**Supplementary Table 4:** **Summary of the final human doses of 13 FLT3 inhibitors.**

| FLT3 inhibitors | CT number | Author | Year | Combined with other drugs or not | Tumor type | Administration regimen |
| --- | --- | --- | --- | --- | --- | --- |
| sorafenib | NCT02576509 | Thomas Yau | 2024 | None | Advanced Hepatocellular Carcinoma | 400 mg sorafenib orally twice daily |
| lestaurtinib | NCT00494585 | M.D. Anderson Cancer Center | 2012 | None | Myelofibrosis | 80 mg orally twice a day for 30 days |
| midostaurin | NCT03280030 | Novartis Pharmaceuticals | 2023 | None | Acute Myeloid Leukemia | Midostaurin 50 mg [two 25 mg capsules] will be administered twice per day by mouth on day 8-21 during induction and consolidation phase; then continuously during continuation phase. |
| gilteritinib | NCT02421939 | Alexander E Perl | 2024 | None | Acute Myeloid Leukemia | Patients received once-daily gilteritinib (120 mg). Gilteritinib was administered in 28-day cycles. |
| quizartinib | NCT02984995 | Daiichi Sankyo Co., Ltd. | 2020 | None | Acute Myeloid Leukemia | Participants who received an initial dose of 30 mg/day of quizartinib and, if no QT prolongation, the dose escalated to 60 mg/day at Day 15. |
| sunitinib | NCT03066427 | Spanish Oncology Genito-Urinary Group | 2020 | None | Clear Cell Renal Carcinoma | Sunitinib 50 mg/day, 4 weeks on/2weeks off |
| crenolanib | NCT01657682 | Allison Galanis | 2023 | None | Acute Myeloid Leukemia | Subjects will take Crenolanib besylate at 100 mg TID until disease progression, death, or unacceptable toxicities. |
| tandutinib | NCT00390468 | National Cancer Institute (NCI) | 2014 | None | Prostate Cancer | 500 mg twice daily, a small-molecule inhibitor of the type III receptor tyrosine kinases. |
| cabozantinib | NCT04804813 | Takeda | 2023 | None | Renal Cell Carcinoma | Cabozantinib 60 milligrams (mg) tablet, orally, once daily for up to 26 weeks. |
| pexidartinib | NCT02975700 | Daiichi Sankyo Co., Ltd. | 2024 | None | Melanoma | 1000 mg/day (400 mg in the morning and 600 mg in the evening) |
| pacritinib | NCT04858256 | University of Michigan Rogel Cancer Center | 2023 | None | T-cell Lymphoproliferative Neoplasms | Pacritinib will be dosed at 200mg twice daily. |
| famitinib | NCT01762293 | Rui-Hua Xu | 2018 | None | Refractory Metastatic Colorectal Cancer | Patients were treated with 25 mg oral famitinib once daily. |
| TAK-659 | NCT03123393 | Calithera Biosciences, Inc | 2023 | None | Diffuse Large B-cell Lymphoma | TAK-659 100 mg tablet, orally, once daily (QD), during each 28-days cycle. |

**Supplementary Table 5 |** **Summary of the target genes of 13 FLT3 inhibitors.**

| FLT3 inhibitors | Gene |
| --- | --- |
| sunitinib | AAK1, ABCB1, ABCG2, BCL2, CD274, CSF1R, EGF, EGFR, ERBB2, FGF2, FLT3, HGF, HIF1A, KDR, KIT, MAPK14, MKI67, PARP1, PDGFRA, PDGFRB, PIK3CD, PTEN, RAF1, RET, STAT3, TNF, TP53, VEGFR1, VEGFR3, VHL |
| quizartinib | ABCB1, ABCG2, AKT1, CBL, FLT3, JAK3, KIT, MYC, Pbx1, STAT3, STAT5 |
| tandutinib | ABCG2, AKT1, FLT3, KIT, MRP7, MTOR, PDGFRA, PDGFRB, STAT3, STAT5 |
| pexidartinib | AIF1, AKT1, CSF1, CSF1R, FLT3, GFAP, KIT, MTOR, PDGFRA, PDGFRB |
| cabozantinib | AKT1, AXL, CDKN1A, FGFR1, FLT3, GAS6, HGF, KIF5B, KIT, MAPK1, MAPK14, MET, MTOR, PDCD1, PDGFRA, PlGF, RET, ROS1, STAT5, TEK, VEGFR1, VEGFR2 |
| Crenolanib | AKT1, CCN2, ETV1, FLT3, HIGD1A, KIT, MAPK1, MTOR, NANOG, PDGFRA, PDGFRB, POU5F1, SHMT2, SLC25A11, SOX2, STAT3, TGFB1, TSC2 |
| gilteritinib | AKT1, AXL, CBL, CRKL, FLT3, MCL1, NFKB1, NPM1, ROS1, STAT5, TIGIT |
| lestaurtinib | AKT1, BCL2L1, FLT3, JAK2, JAK3, MAPK1, MAST1, MYC, NFKB1, NGF, NTRK1, NTRK2, PKN1, PTPN6, STAT3, STAT5, TYK2 |
| midostaurin | AKT1, BCL2, CBL, FLT3, JUN, KIT, MAPK8, MTOR, NFKB1, PDGFRA, PDGFRB, STAT5, SYK, VEGFR2 |
| sorafenib | AKT1, BCL2L1, BRAF, CBL, CDK4, CDKN1A, EGF, EGFR, FGFR1, FLT3, HGF, HIF1A, JAK2, KIT, MAPK1, MAPK14, MAPK8, MCL1, MEK, MET, MKI67, MTOR, NFE2L2, PDGFRB, PIK3CD, PRKAA1, PTPN6, RAF1, SLC7A11, STAT3, VEGFR2, VEGFR3 |
| TAK-659 | BTK, FLT3, SYK |
| Pacritinib | CSF1R, FGFR1, FLT3, IRAK1, JAK1, JAK2, JAK3, MTOR, S100A9, STAT3, STAT5 |
| Famitinib | FLT3, RET, VEGFR2, VEGFR3, KIT, PDGFRA, PDGFRB, VEGFA, VEGFR3, VEGFR2, CD8, CYP3A4, KAT6A, VEGFC, VEGFR1, CYP3A5, FLT3, CD274, CYP1A1, CYP1A2, BRAF, HIF1A, ERBB2, IGF1, PTH, EGFR, AKT1 |

**Supplementary Table 6 | Published articles related to the target genes of FLT3 inhibitors.**

| FLT3 inhibitors | Gene | DOI / PMID | Title |
| --- | --- | --- | --- |
| sunitinib | AAK1 | DOI: 10.1128/JVI.02705-14 | AP-2-Associated Protein Kinase 1 and Cyclin G-Associated Kinase Regulate Hepatitis C Virus Entry and Are Potential Drug Targets |
| quizartinib | ABCB1 | DOI: 10.1371/journal.pone.0071266 | The FLT3 inhibitor quizartinib inhibits ABCG2 at pharmacologically relevant concentrations, with implications for both chemosensitization and adverse drug interactions |
| sunitinib | ABCB1 | DOI: 10.1124/dmd.108.024612 | Sunitinib (Sutent, SU11248), a small-molecule receptor tyrosine kinase inhibitor, blocks function of the ATP-binding cassette (ABC) transporters P-glycoprotein (ABCB1) and ABCG2 |
| quizartinib | ABCG2 | DOI: 10.1371/journal.pone.0071266 | The FLT3 inhibitor quizartinib inhibits ABCG2 at pharmacologically relevant concentrations, with implications for both chemosensitization and adverse drug interactions |
| sunitinib | ABCG2 | DOI: 10.1124/dmd.108.024612 | Sunitinib (Sutent, SU11248), a small-molecule receptor tyrosine kinase inhibitor, blocks function of the ATP-binding cassette (ABC) transporters P-glycoprotein (ABCB1) and ABCG2 |
| tandutinib | ABCG2 | DOI: 10.1016/j.ejps.2013.04.015 | Tandutinib (MLN518/CT53518) targeted to stem-like cells by inhibiting the function of ATP-binding cassette subfamily G member 2 |
| pexidartinib | AIF1 | PMID: 32642296 | Inflammation suppression prevents tumor cell proliferation in a mouse model of thyroid cancer |
| cabozantinib | AKT1 | DOI: 10.1155/2020/1649453 | Activating CD137 Signaling Promotes Sprouting Angiogenesis via Increased VEGFA Secretion and the VEGFR2/Akt/eNOS Pathway |
| Crenolanib | AKT1 | DOI: 10.1371/journal.pone.0172191 | The platelet-derived growth factor receptor/STAT3 signaling pathway regulates the phenotypic transition of corpus cavernosum smooth muscle in rats |
| gilteritinib | AKT1 | DOI: 10.1111/jcmm.14913 | Gilteritinib induces PUMA-dependent apoptotic cell death via AKT/GSK-3β/NF-κB pathway in colorectal cancer cells |
| lestaurtinib | AKT1 | DOI: 10.1038/s41388-022-02475-8 | CRISPR-based kinome-screening revealed MINK1 as a druggable player to rewire 5FU-resistance in OSCC through AKT/MDM2/p53 axis |
| midostaurin | AKT1 | DOI: 10.1186/s12885-022-09828-2 | Combination of midostaurin and ATRA exerts dose-dependent dual effects on acute myeloid leukemia cells with wild type FLT3 |
| pexidartinib | AKT1 | DOI: 10.1158/1078-0432.CCR-19-1486 | Colony-Stimulating Factor 1 Receptor (CSF1R) Activates AKT/mTOR Signaling and Promotes T-Cell Lymphoma Viability |
| quizartinib | AKT1 | DOI: 10.1016/j.bcp.2021.114538 | Homoharringtonine synergizes with quizartinib in FLT3-ITD acute myeloid leukemia by targeting FLT3-AKT-c-Myc pathway |
| sorafenib | AKT1 | PMCID: PMC2872747 | Sorafenib downregulates ERK/Akt and STAT3 survival pathways and induces apoptosis in a human neuroblastoma cell line |
| tandutinib | AKT1 | DOI: 10.1158/1535-7163.MCT-12-0907 | Tandutinib inhibits the Akt/mTOR signaling pathway to inhibit colon cancer growth |
| cabozantinib | AXL | DOI: 10.1056/NEJMoa1717002 | Cabozantinib in Patients with Advanced and Progressing Hepatocellular Carcinoma |
| gilteritinib | AXL | DOI: 10.1007/S10637-017-0470-Z | Gilteritinib, a FLT3/AXL inhibitor, shows antileukemic activity in mouse models of FLT3 mutated acute myeloid leukemia |
| midostaurin | BCL2 | DOI: 10.19746/j.cnki.issn.1009-2137.2021.01.010 | [Inhibitory Effect of PKC412 Against Human Acute Leukemia Cell Line HL-60 Cells] |
| sunitinib | BCL2 | DOI: 10.3390/ph13110397 | Small-Dose Sunitinib Modulates p53, Bcl-2, STAT3, and ERK1/2 Pathways and Protects against Adenine-Induced Nephrotoxicity |
| lestaurtinib | BCL2L1 | DOI: 10.1371/journal.pone.0018856 | Lestaurtinib inhibition of the Jak/STAT signaling pathway in hodgkin lymphoma inhibits proliferation and induces apoptosis |
| sorafenib | BCL2L1 | DOI: 10.4161/cbt.8.18.9208 | Sorafenib induces growth inhibition and apoptosis in human synovial sarcoma cells via inhibiting the RAF/MEK/ERK signaling pathway |
| sorafenib | BRAF | PMID: 19542731 | [Sorafenib(Nexavar)] |
| TAK-659 | BTK | DOI: 10.1152/ajpcell.00296.2020 | Assessment of the effects of Syk and BTK inhibitors on GPVI-mediated platelet signaling and function |
| gilteritinib | CBL | DOI: 10.1111/bjh.16092 | Comparison of effects of midostaurin, crenolanib, quizartinib, gilteritinib, sorafenib and BLU-285 on oncogenic mutants of KIT, CBL and FLT3 in haematological malignancies |
| midostaurin | CBL | DOI: 10.1111/bjh.16092 | Comparison of effects of midostaurin, crenolanib, quizartinib, gilteritinib, sorafenib and BLU-285 on oncogenic mutants of KIT, CBL and FLT3 in haematological malignancies |
| quizartinib | CBL | DOI: 10.1111/bjh.16092 | Comparison of effects of midostaurin, crenolanib, quizartinib, gilteritinib, sorafenib and BLU-285 on oncogenic mutants of KIT, CBL and FLT3 in haematological malignancies |
| sorafenib | CBL | DOI: 10.1080/16078454.2023.2204620 | Sorafenib regulates c-CBL gene-mediated chemoresistance in acute myeloid leukemia cells突变+耐药 |
| Crenolanib | CCN2 | DOI: 10.1016/j.jid.2017.03.032 | Blockade of PDGF Receptors by Crenolanib Has Therapeutic Effect in Patient Fibroblasts and in Preclinical Models of Systemic Sclerosis |
| sunitinib | CD274 | DOI: 10.2217/fon-2019-0725 | Sunitinib inhibits PD-L1 expression in osteosarcoma by targeting STAT3 and remodels the immune system in tumor-bearing mice |
| sorafenib | CDK4 | DOI： 10.1111/jcmm.15795 | Pterostilbene enhances sorafenib's anticancer effects on gastric adenocarcinoma |
| cabozantinib | CDKN1A | DOI: 10.1136/GUTJNL-2020-320716 | Cabozantinib-based combination therapy for the treatment of hepatocellular carcinoma |
| sorafenib | CDKN1A | DOI: 10.1093/jpp/rgaa053 | Sorafenib suppresses proliferation rate of fibroblast-like synoviocytes through the arrest of cell cycle in experimental adjuvant arthritis |
| gilteritinib | CRKL | DOI: 10.1182/blood.2021012976 | Genome-wide CRISPR-Cas9 screen identifies rationally designed combination therapies for CRLF2-rearranged Ph-like ALL |
| pexidartinib | CSF1 | DOI: 10.1158/1535-7163.MCT-20-0591 | CSF1/CSF1R Signaling Inhibitor Pexidartinib (PLX3397) Reprograms Tumor-Associated Macrophages and Stimulates T-cell Infiltration in the Sarcoma Microenvironment |
| Pacritinib | CSF1R | DOI: 10.1358/dot.2022.58.12.3474538 | Pacritinib for myelofibrosis in adults with thrombocytopenia |
| pexidartinib | CSF1R | DOI: 10.2174/1871520620999201102123555 | Prospects of Treating Tenosynovial Giant Cell Tumor through Pexidartinib: A Review |
| sunitinib | CSF1R | DOI: 10.2165/11318860-000000000-00000 | Sunitinib: a multitargeted receptor tyrosine kinase inhibitor in the era of molecular cancer therapies |
| sorafenib | EGF | DOI: 10.3892/mmr.2017.6773 | Sorafenib controls the epithelial‑mesenchymal transition of ovarian cancer cells via EGF and the CD44‑HA signaling pathway in a cell type‑dependent manner |
| sunitinib | EGF | DOI: 10.1016/j.molonc.2012.06.006 | Blockade of NFκB activity by Sunitinib increases cell death in Bortezomib-treated endometrial carcinoma cells |
| sorafenib | EGFR | DOI: 10.1016/j.cellsig.2014.01.026 | Upregulation of HIF-2α induced by sorafenib contributes to the resistance by activating the TGF-α/EGFR pathway in hepatocellular carcinoma cells |
| sunitinib | EGFR | DOI: 10.1124/jpet.115.226639 | Induction of epithelial-mesenchymal transition via activation of epidermal growth factor receptor contributes to sunitinib resistance in human renal cell carcinoma cell lines |
| sunitinib | ERBB2 | DOI: 10.1007/s00262-022-03146-z | The multitargeted receptor tyrosine kinase inhibitor sunitinib induces resistance of HER2 positive breast cancer cells to trastuzumab-mediated ADCC |
| Crenolanib | ETV1 | DOI: 10.1053/j.gastro.2015.04.006 | Platelet-Derived Growth Factor Receptor-α Regulates Proliferation of Gastrointestinal Stromal Tumor Cells With Mutations in KIT by Stabilizing ETV1 |
| sunitinib | FGF2 | DOI: 10.1007/s11523-014-0305-1 | Sunitinib administered prior to radiotherapy in patients with non-resectable glioblastoma: results of a phase II study |
| cabozantinib | FGFR1 | DOI: 10.3390/cancers12010244 | Resistance to MET/VEGFR2 Inhibition by Cabozantinib Is Mediated by YAP/TBX5-Dependent Induction of FGFR1 in Castration-Resistant Prostate Cancer |
| Pacritinib | FGFR1 | DOI: 10.1186/s12943-021-01460-1 | IRAK1-regulated IFN-γ signaling induces MDSC to facilitate immune evasion in FGFR1-driven hematological malignancies |
| sorafenib | FGFR1 | DOI: 10.1186/s13287-017-0573-7 | The inhibition of FGF receptor 1 activity mediates sorafenib antiproliferative effects in human malignant pleural mesothelioma tumor-initiating cells |
| cabozantinib | FLT3 | DOI: 10.1186/s13045-018-0675-4 | FLT3 inhibitors in acute myeloid leukemia |
| Crenolanib | FLT3 | DOI: 10.1186/s13045-018-0675-4 | FLT3 inhibitors in acute myeloid leukemia |
| Famitinib | FLT3 | PMID: 24238512 | Famitinib in metastatic renal cell carcinoma: a single center study |
| gilteritinib | FLT3 | DOI: 10.1200/JCO.22.00602 | Venetoclax Plus Gilteritinib for FLT3-Mutated Relapsed/Refractory Acute Myeloid Leukemia |
| lestaurtinib | FLT3 | DOI: 10.1186/s13045-018-0675-4 | FLT3 inhibitors in acute myeloid leukemia |
| midostaurin | FLT3 | DOI: 10.1186/s13045-018-0675-4 | FLT3 inhibitors in acute myeloid leukemia |
| Pacritinib | FLT3 | DOI: 10.1038/s41598-023-28576-2 | Pacritinib inhibits glucose consumption in squamous cell lung cancer cells by targeting FLT3 |
| pexidartinib | FLT3 | DOI: 10.1158/1078-0432.CCR-20-3458 | Recurrent Mutations in Cyclin D3 Confer Clinical Resistance to FLT3 Inhibitors in Acute Myeloid Leukemia |
| quizartinib | FLT3 | DOI: 10.1186/s13045-018-0675-4 | FLT3 inhibitors in acute myeloid leukemia |
| sorafenib | FLT3 | DOI: 10.1186/s13045-018-0675-4 | FLT3 inhibitors in acute myeloid leukemia |
| sunitinib | FLT3 | DOI: 10.1186/s13045-018-0675-4 | FLT3 inhibitors in acute myeloid leukemia |
| TAK-659 | FLT3 | DOI: 10.1002/pbc.30503 | In vivo activity of the dual SYK/FLT3 inhibitor TAK-659 against pediatric acute lymphoblastic leukemia xenografts |
| tandutinib | FLT3 | DOI: 10.1186/s13045-018-0675-4 | FLT3 inhibitors in acute myeloid leukemia |
| cabozantinib | GAS6 | DOI: 10.1038/S41416-021-01559-8 | Landscape of prognostic signatures and immunogenomics of the AXL/GAS6 axis in renal cell carcinoma |
| pexidartinib | GFAP | DOI: 10.1186/s12974-021-02118-X | Pexidartinib treatment in Alexander disease model mice reduces macrophage numbers and increases glial fibrillary acidic protein levels, yet has minimal impact on other disease phenotypes |
| cabozantinib | HGF | DOI: 10.1016/j.critrevonc.2021.103234 | Combination of HGF/MET-targeting agents and other therapeutic strategies in cancer |
| sorafenib | HGF | DOI: 10.1016/j.ejrad.2011.04.042 | Multikinase inhibitor sorafenib transiently promotes necrosis after radiofrequency ablation in rat liver but activates growth signals |
| sunitinib | HGF | DOI: 10.1136/jitc-2020-001038 | Synergizing sunitinib and radiofrequency ablation to treat hepatocellular cancer by triggering the antitumor immune response |
| sorafenib | HIF1A | DOI: 10.1111/cpr.13158 | Sorafenib attenuates liver fibrosis by triggering hepatic stellate cell ferroptosis via HIF-1α/SLC7A11 pathway |
| sunitinib | HIF1A | DOI: 10.1016/j.bbrc.2010.06.060 | Sunitinib deregulates tumor adaptation to hypoxia by inhibiting HIF-1alpha synthesis in HT-29 colon cancer cells |
| Crenolanib | HIGD1A | DOI: 10.1002/cbic.201900067 | Crenolanib-Derived Probes Suitable for Cell- and Tissue-Based Protein Profiling and Single-Cell Imaging |
| Pacritinib | IRAK1 | DOI: 10.1182/bloodadvances.2023010151 | Pacritinib is a potent ACVR1 inhibitor with significant anemia benefit in patients with myelofibrosis |
| Pacritinib | JAK1 | DOI: 10.3324/haematol.2022.282612 | Momelotinib (JAK1/JAK2/ACVR1 inhibitor): mechanism of action, clinical trial reports, and therapeutic prospects beyond myelofibrosis |
| lestaurtinib | JAK2 | DOI: 10.1371/journal.pone.0018856 | Lestaurtinib inhibition of the Jak/STAT signaling pathway in hodgkin lymphoma inhibits proliferation and induces apoptosis |
| Pacritinib | JAK2 | DOI: 10.1002/hep.32746 | Janus kinase 2 inhibition by pacritinib as potential therapeutic target for liver fibrosis |
| sorafenib | JAK2 | DOI: 10.3389/fcell.2021.660005 | Oxidative Stress Activated by Sorafenib Alters the Temozolomide Sensitivity of Human Glioma Cells Through Autophagy and JAK2/STAT3-AIF Axis |
| lestaurtinib | JAK3 | DOI: 10.1016/j.phrs.2016.07.038 | Janus kinase (JAK) inhibitors in the treatment of inflammatory and neoplastic diseases |
| Pacritinib | JAK3 | DOI: 10.2174/1871520623666230320120915 | First Approval of Pacritinib as a Selective Janus Associated Kinase-2 Inhibitor for the Treatment of Patients with Myelofibrosis |
| quizartinib | JAK3 | DOI： 10.1038/s41598-020-73020-4 | High-fat diet intensifies MLL-AF9-induced acute myeloid leukemia through activation of the FLT3 signaling in mouse primitive hematopoietic cells |
| midostaurin | JUN | DOI: 10.1016/j.bbrc.2007.06.009 | PKC412 (CGP41251) modulates the proliferation and lipopolysaccharide-induced inflammatory responses of RAW 264.7 macrophages |
| sunitinib | KDR | DOI: 10.1002/2211-5463.13399 | CRISPR-mediated knockout of VEGFR2/KDR inhibits cell growth in a squamous thyroid cancer cell line |
| cabozantinib | KIF5B | DOI: 10.1158/1535-7163.MCT-16-0258 | Preclinical Modeling of KIF5B-RET Fusion Lung Adenocarcinoma |
| cabozantinib | KIT | DOI: 10.2217/FON-2016-0358 | Cabozantinib in genitourinary malignancies |
| Crenolanib | KIT | DOI: 10.1182/blood-2013-10-529313 | Crenolanib is a potent inhibitor of FLT3 with activity against resistance-conferring point mutants |
| midostaurin | KIT | DOI: 10.1182/blood.2019000932 | New developments in diagnosis, prognostication, and treatment of advanced systemic mastocytosis |
| pexidartinib | KIT | DOI: 10.1007/s40265-019-01210-0 | Pexidartinib: First Approval |
| quizartinib | KIT | DOI: 10.1186/1476-4598-12-19 | Quizartinib (AC220) is a potent second generation class III tyrosine kinase inhibitor that displays a distinct inhibition profile against mutant-FLT3, -PDGFRA and -KIT isoforms |
| sorafenib | KIT | DOI: 10.1111/bjh.16092 | Comparison of effects of midostaurin, crenolanib, quizartinib, gilteritinib, sorafenib and BLU-285 on oncogenic mutants of KIT, CBL and FLT3 in haematological malignancies |
| sunitinib | KIT | DOI: 10.2174/0929867324666171006165942 | Sunitinib in the Treatment of Thyroid Cancer |
| tandutinib | KIT | DOI: 10.1158/1535-7163.MCT-12-0907 | Tandutinib inhibits the Akt/mTOR signaling pathway to inhibit colon cancer growth |
| cabozantinib | MAPK1 | DOI:10.1136/gutjnl-2020-320716 | Cabozantinib-based combination therapy for the treatment of hepatocellular carcinoma |
| Crenolanib | MAPK1 | DOI: 10.1002/cbic.201900067 | Crenolanib-Derived Probes Suitable for Cell- and Tissue-Based Protein Profiling and Single-Cell Imaging |
| lestaurtinib | MAPK1 | DOI： 10.1038/leu.2012.191 | Mutations of FLT3/ITD confer resistance to multiple tyrosine kinase inhibitors |
| sorafenib | MAPK1 | DOI: 10.1016/j.etap.2014.07.012 | Sorafenib regulating ERK signals pathway in gastric cancer cell |
| cabozantinib | MAPK14 | DOI: 10.1038/S41417-021-00358-W | Cabozantinib promotes erythroid differentiation in K562 erythroleukemia cells through global changes in gene expression and JNK activation |
| sorafenib | MAPK14 | DOI: 10.1038/S41420-022-01073-7 | Sorafenib inhibits LPS-induced inflammation by regulating Lyn-MAPK-NF-kB/AP-1 pathway and TLR4 expression |
| sunitinib | MAPK14 | DOI: 10.1111/odi.14457 | Sunitinib promotes apoptosis via p38 MAPK activation and STAT3 downregulation in oral keratinocytes |
| midostaurin | MAPK8 | DOI: 10.1182/blood-2006-05-014092 | PKC412 demonstrates JNK-dependent activity against human multiple myeloma cells |
| sorafenib | MAPK8 | DOI: 10.1016/j.bcp.2019.113728 | JNK activation and translocation to mitochondria mediates mitochondrial dysfunction and cell death induced by VDAC opening and sorafenib in hepatocarcinoma cells |
| lestaurtinib | MAST1 | DOI: 10.1016/j.ccell.2018.06.012 | MAST1 Drives Cisplatin Resistance in Human Cancers by Rewiring cRaf-Independent MEK Activation |
| gilteritinib | MCL1 | DOI: 10.1038/s41392-021-00578-4 | FLT3 tyrosine kinase inhibitors synergize with BCL-2 inhibition to eliminate FLT3/ITD acute leukemia cells through BIM activation |
| sorafenib | MCL1 | DOI: 10.1016/j.neo.2015.07.003 | Sorafenib Sensitizes Glioma Cells to the BH3 Mimetic ABT-737 by Targeting MCL1 in a STAT3-Dependent Manner |
| sorafenib | MEK | DOI: 10.1093/carcin/bgy038 | Sorafenib inhibits proliferation and invasion in desmoid-derived cells by targeting Ras/MEK/ERK and PI3K/Akt/mTOR pathways |
| cabozantinib | MET | DOI: 10.2217/FON-2016-0358 | Cabozantinib in genitourinary malignancies |
| sorafenib | MET | DOI: 10.1002/hed.25440 | Receptor tyrosine kinase MET as potential target of multi-kinase inhibitor and radiosensitizer sorafenib in HNSCC |
| sorafenib | MKI67 | DOI: 10.1186/S13287-022-02888-Y | Sorafenib targets and inhibits the oncogenic properties of endometrial cancer stem cells via the RAF/ERK pathway |
| sunitinib | MKI67 | DOI: 10.1016/j.urolonc.2008.03.017 | Sunitinib malate is active against human urothelial carcinoma and enhances the activity of cisplatin in a preclinical model |
| tandutinib | MRP7 | DOI: 10.3892/or.2013.2362 | andutinib (MLN518) reverses multidrug resistance by inhibiting the efflux activity of the multidrug resistance protein 7 (ABCC10) |
| cabozantinib | MTOR | DOI： 10.1038/S41417-021-00329-1 | Repurposing cabozantinib with therapeutic potential in KIT-driven t(8;21) acute myeloid leukaemias |
| Crenolanib | MTOR | DOI: 10.1158/1541-7786.MCR-20-0600 | Crenolanib Regulates ERK and AKT/mTOR Signaling Pathways in RAS/BRAF-Mutated Colorectal Cancer Cells and Organoids |
| midostaurin | MTOR | DOI: 10.1002/ijc.32081 | The multikinase inhibitor EC-70124 synergistically increased the antitumor activity of doxorubicin in sarcomas |
| Pacritinib | MTOR | DOI: 10.1158/1078-0432.CCR-20-4725 | Pacritinib Combined with Sirolimus and Low-Dose Tacrolimus for GVHD Prevention after Allogeneic Hematopoietic Cell Transplantation: Preclinical and Phase I Trial Results |
| pexidartinib | MTOR | DOI: 10.1158/1078-0432.CCR-19-1486 | Colony-Stimulating Factor 1 Receptor (CSF1R) Activates AKT/mTOR Signaling and Promotes T-Cell Lymphoma Viability |
| sorafenib | MTOR | DOI: 10.1096/fj.201802619RR | Sorafenib kills liver cancer cells by disrupting SCD1-mediated synthesis of monounsaturated fatty acids via the ATP-AMPK-mTOR-SREBP1 signaling pathway |
| tandutinib | MTOR | DOI: 10.1158/1535-7163.MCT-12-0907 | Tandutinib inhibits the Akt/mTOR signaling pathway to inhibit colon cancer growth |
| lestaurtinib | MYC | DOI: 10.1016/j.crtox.2022.100102 | Lestaurtinib induces DNA damage that is related to estrogen receptor activation |
| quizartinib | MYC | DOI: 10.1016/j.bcp.2021.114538 | Homoharringtonine synergizes with quizartinib in FLT3-ITD acute myeloid leukemia by targeting FLT3-AKT-c-Myc pathway |
| Crenolanib | NANOG | DOI: 10.1158/1541-7786.MCR-20-0600 | Crenolanib Regulates ERK and AKT/mTOR Signaling Pathways in RAS/BRAF-Mutated Colorectal Cancer Cells and Organoids |
| sorafenib | NFE2L2 | DOI: 10.1002/hep.28251 | Activation of the p62-Keap1-NRF2 pathway protects against ferroptosis in hepatocellular carcinoma cells |
| gilteritinib | NFKB1 | DOI: 10.1111/jcmm.14913 | Gilteritinib induces PUMA-dependent apoptotic cell death via AKT/GSK-3β/NF-κB pathway in colorectal cancer cells |
| lestaurtinib | NFKB1 | DOI: 10.18632/oncotarget.13100 | Investigating the mechanism of hepatocellular carcinoma progression by constructing genetic and epigenetic networks using NGS data identification and big database mining method |
| midostaurin | NFKB1 | DOI: 10.1038/s41419-018-1259-5 | Midostaurin potentiates rituximab antitumor activity in Burkitt's lymphoma by inducing apoptosis |
| lestaurtinib | NGF | DOI: 10.18632/oncotarget.3227 | NGF-induced TrkA/CD44 association is involved in tumor aggressiveness and resistance to lestaurtinib |
| gilteritinib | NPM1 | DOI: 10.1158/1541-7786.MCR-20-0738 | Preclinical Evaluation of Gilteritinib on NPM1-ALK-Driven Anaplastic Large Cell Lymphoma Cells |
| lestaurtinib | NTRK1 | DOI: 10.1158/1535-7163.MCT-12-0532 | Induction of endoplasmic reticulum stress by sorafenib and activation of NF-κB by lestaurtinib as a novel resistance mechanism in Hodgkin lymphoma cell lines |
| lestaurtinib | NTRK2 | DOI: 10.18632/oncotarget.13100 | Investigating the mechanism of hepatocellular carcinoma progression by constructing genetic and epigenetic networks using NGS data identification and big database mining method |
| sunitinib | PARP1 | DOI: 10.1016/j.eururo.2022.05.013 | Interrogating the Significance of PARP1 Expression and PBRM1 Mutation as Biomarkers for Predicting the Response to Atezolizumab plus Bevacizumab or to Sunitinib in Patients with Clear Cell Renal Cell Carcinoma |
| quizartinib | Pbx1 | DOI: 10.1371/journal.pone.0158290 | Internal Tandem Duplication in FLT3 Attenuates Proliferation and Regulates Resistance to the FLT3 Inhibitor AC220 by Modulating p21Cdkn1a and Pbx1 in Hematopoietic Cells |
| cabozantinib | PDCD1 | DOI: 10.2217/fon-2022-0802 | LITESPARK-011: belzutifan plus lenvatinib vs cabozantinib in advanced renal cell carcinoma after anti-PD-1/PD-L1 therapy |
| cabozantinib | PDGFRA | DOI: 10.1093/noajnl/vdab179 | Phase I study using crenolanib to target PDGFR kinase in children and young adults with newly diagnosed DIPG or recurrent high-grade glioma, including DIPG |
| Crenolanib | PDGFRA | DOI: 10.1158/1078-0432.CCR-12-0625 | Crenolanib inhibits the drug-resistant PDGFRA D842V mutation associated with imatinib-resistant gastrointestinal stromal tumors |
| midostaurin | PDGFRA | DOI: 10.1007/s11899-015-0280-3 | Tyrosine Kinase Inhibitors and Therapeutic Antibodies in Advanced Eosinophilic Disorders and Systemic Mastocytosis |
| pexidartinib | PDGFRA | DOI: 10.1007/s40265-019-01210-0 | Pexidartinib: First Approval |
| sunitinib | PDGFRA | DOI: 10.3390/biomedicines10112842 | Whole Exome Sequencing Identifies PHF14 Mutations in Neurocytoma and Predicts Responsivity to the PDGFR Inhibitor Sunitinib |
| tandutinib | PDGFRA | DOI: 10.1097/MPH.0b013e3182309fe4 | Preclinical testing of tandutinib in a transgenic medulloblastoma mouse model |
| Crenolanib | PDGFRB | DOI: 10.1002/cbic.201900067 | Crenolanib-Derived Probes Suitable for Cell- and Tissue-Based Protein Profiling and Single-Cell Imaging |
| midostaurin | PDGFRB | DOI: 10.1358/dot.2017.53.10.2717625 | Midostaurin for the treatment of adult patients with newly diagnosed acute myeloid leukemia that is FLT3 mutation-positive |
| pexidartinib | PDGFRB | DOI: 10.1158/1078-0432.CCR-13-2576 | Sustained inhibition of receptor tyrosine kinases and macrophage depletion by PLX3397 and rapamycin as a potential new approach for the treatment of MPNSTs |
| sorafenib | PDGFRB | DOI: 10.1517/14656566.7.4.453 | Sorafenib |
| sunitinib | PDGFRB | DOI: 10.2174/0929867324666171006165942 | Sunitinib in the Treatment of Thyroid Cancer |
| tandutinib | PDGFRB | DOI: 10.1093/neuonc/now185 | Feasibility, phase I, and phase II studies of tandutinib, an oral platelet-derived growth factor receptor-β tyrosine kinase inhibitor, in patients with recurrent glioblastoma |
| sorafenib | PIK3CD | DOI: 10.1186/s13046-022-02296-3 | Macropinocytosis is an alternative pathway of cysteine acquisition and mitigates sorafenib-induced ferroptosis in hepatocellular carcinoma |
| sunitinib | PIK3CD | DOI： 10.1155/2022/6042518 | Sunitinib Reduced the Migration of Ectopic Endometrial Cells via p-VEGFR-PI3K-AKT-YBX1-Snail Signaling Pathway |
| lestaurtinib | PKN1 | DOI: 10.1371/journal.pone.0034973 | Lestaurtinib inhibits histone phosphorylation and androgen-dependent gene expression in prostate cancer cells |
| cabozantinib | PlGF | DOI: 10.1007/s10549-019-05445-z | A phase II study of cabozantinib alone or in combination with trastuzumab in breast cancer patients with brain metastases |
| Crenolanib | POU5F1 | DOI: 10.1158/1541-7786.MCR-20-0600 | Crenolanib Regulates ERK and AKT/mTOR Signaling Pathways in RAS/BRAF-Mutated Colorectal Cancer Cells and Organoids |
| sorafenib | PRKAA1 | DOI: 10.1016/j.molmet.2023.101796 | Glycolysis maintains AMPK activation in sorafenib-induced Warburg effect |
| sunitinib | PTEN | DOI: 10.1007/s11060-010-0259-9 | Sunitinib induces PTEN expression and inhibits PDGFR signaling and migration of medulloblastoma cells |
| lestaurtinib | PTPN6 | DOI: 10.1074/JBC。M411974200 | FLT3/ITD mutation signaling includes suppression of SHP-1 |
| sorafenib | PTPN6 | DOI: 10.3390/ijms25010331 | Consideration of SHP-1 as a Molecular Target for Tumor Therapy |
| sorafenib | RAF1 | DOI: 10.2146/ajhp060661 | Role of sunitinib and sorafenib in the treatment of metastatic renal cell carcinoma |
| sunitinib | RAF1 | DOI: 10.1111/CAS.12176 | Sunitinib induces cellular senescence via p53/Dec1 activation in renal cell carcinoma cells |
| cabozantinib | RET | DOI: 10.2217/FON-2016-0358 | Cabozantinib in genitourinary malignancies |
| Famitinib | RET | PMID: 24238512 | Famitinib in metastatic renal cell carcinoma: a single center study |
| sunitinib | RET | DOI: 10.2174/0929867324666171006165942 | Sunitinib in the Treatment of Thyroid Cancer |
| cabozantinib | ROS1 | DOI: 10.1158/1078-0432.CCR-15-1601 | Identification of Existing Drugs That Effectively Target NTRK1 and ROS1 Rearrangements in Lung Cancer |
| gilteritinib | ROS1 | DOI: 10.1101/2024.01.16.575901 | TKI Type Switching Overcomes ROS1 L2086F in ROS1 Fusion-Positive Cancers |
| Pacritinib | S100A9 | DOI: 10.1158/0008-5472.CAN-20-2125 | Targeting the IRAK1-S100A9 Axis Overcomes Resistance to Paclitaxel in Nasopharyngeal Carcinoma |
| Crenolanib | SHMT2 | DOI: 10.1002/cbic.201900067 | Crenolanib-Derived Probes Suitable for Cell- and Tissue-Based Protein Profiling and Single-Cell Imaging |
| Crenolanib | SLC25A11 | DOI: 10.1002/cbic.201900067 | Crenolanib-Derived Probes Suitable for Cell- and Tissue-Based Protein Profiling and Single-Cell Imaging |
| sorafenib | SLC7A11 | DOI: 10.1111/cpr.13158 | Sorafenib attenuates liver fibrosis by triggering hepatic stellate cell ferroptosis via HIF-1α/SLC7A11 pathway |
| Crenolanib | SOX2 | DOI: 10.1158/1541-7786.MCR-20-0600 | Crenolanib Regulates ERK and AKT/mTOR Signaling Pathways in RAS/BRAF-Mutated Colorectal Cancer Cells and Organoids |
| Crenolanib | STAT3 | DOI: 10.1371/journal.pone.0172191 | The platelet-derived growth factor receptor/STAT3 signaling pathway regulates the phenotypic transition of corpus cavernosum smooth muscle in rats |
| lestaurtinib | STAT3 | DOI: 10.1371/journal.pone.0018856 | Lestaurtinib inhibition of the Jak/STAT signaling pathway in hodgkin lymphoma inhibits proliferation and induces apoptosis |
| Pacritinib | STAT3 | DOI: 10.1371/journal.pone.0189670 | The JAK2/STAT3 inhibitor pacritinib effectively inhibits patient-derived GBM brain tumor initiating cells in vitro and when used in combination with temozolomide increases survival in an orthotopic xenograft model |
| quizartinib | STAT3 | DOI: 10.1038/s41598-020-73020-4 | High-fat diet intensifies MLL-AF9-induced acute myeloid leukemia through activation of the FLT3 signaling in mouse primitive hematopoietic cells |
| sorafenib | STAT3 | DOI: 10.3389/fcell.2021.660005 | Oxidative Stress Activated by Sorafenib Alters the Temozolomide Sensitivity of Human Glioma Cells Through Autophagy and JAK2/STAT3-AIF Axis |
| sunitinib | STAT3 | DOI: 10.1111/odi.14457 | Sunitinib promotes apoptosis via p38 MAPK activation and STAT3 downregulation in oral keratinocytes |
| tandutinib | STAT3 | DOI: 10.1182/blood-2004-06-2189 | Sensitivity of oncogenic KIT mutants to the kinase inhibitors MLN518 and PD180970 |
| cabozantinib | STAT5 | DOI: 10.3390/ijms20051230 | Cytarabine-Resistant FLT3-ITD Leukemia Cells are Associated with TP53 Mutation and Multiple Pathway Alterations-Possible Therapeutic Efficacy of Cabozantinib |
| gilteritinib | STAT5 | DOI: 10.1016/j.tranon.2022.101354 | Inhibition of BCL2A1 by STAT5 inactivation overcomes resistance to targeted therapies of FLT3-ITD/D835 mutant AML |
| lestaurtinib | STAT5 | DOI: 10.1371/journal.pone.0018856 | Lestaurtinib inhibition of the Jak/STAT signaling pathway in hodgkin lymphoma inhibits proliferation and induces apoptosis |
| midostaurin | STAT5 | DOI: 10.1182/blood-2007-07-101238 | Uniform sensitivity of FLT3 activation loop mutants to the tyrosine kinase inhibitor midostaurin |
| Pacritinib | STAT5 | DOI: 10.1038/leu.2011.148 | SB1518, a novel macrocyclic pyrimidine-based JAK2 inhibitor for the treatment of myeloid and lymphoid malignancies |
| quizartinib | STAT5 | DOI: 10.19746/j.cnki.issn.1009-2137.2022.03.003 | [Effects of Paclitaxel and Quizartinib Alone and in Combination on AML Cell Line MV4-11 and Its STAT5 Signal Pathway] |
| tandutinib | STAT5 | DOI: 10.1182/blood-2003-12-4446 | Variable sensitivity of FLT3 activation loop mutations to the small molecule tyrosine kinase inhibitor MLN518 |
| midostaurin | SYK | DOI: 10.18632/oncotarget.19036 | Characterization of midostaurin as a dual inhibitor of FLT3 and SYK and potentiation of FLT3 inhibition against FLT3-ITD-driven leukemia harboring activated SYK kinase |
| TAK-659 | SYK | DOI: 10.1002/pbc.30503 | In vivo activity of the dual SYK/FLT3 inhibitor TAK-659 against pediatric acute lymphoblastic leukemia xenografts |
| cabozantinib | TEK | DOI: 10.2174/1574892810666150708110816 | Cabozantinib in Thyroid Cancer |
| Crenolanib | TGFB1 | DOI： 10.1158/1541-7786.MCR-20-0600 | Crenolanib Regulates ERK and AKT/mTOR Signaling Pathways in RAS/BRAF-Mutated Colorectal Cancer Cells and Organoids |
| gilteritinib | TIGIT | DOI: 10.1038/S41409-022-01619-4 | Gilteritinib enhances graft-versus-leukemia effects against FLT3-ITD mutant leukemia after allogeneic hematopoietic stem cell transplantation |
| sunitinib | TNF | DOI: 10.1016/j.antiviral.2018.07.022 | Synergism between the tyrosine kinase inhibitor sunitinib and Anti-TNF antibody protects against lethal dengue infection |
| sunitinib | TP53 | DOI: 10.1111/cas.12176 | Sunitinib induces cellular senescence via p53/Dec1 activation in renal cell carcinoma cells |
| Crenolanib | TSC2 | DOI: 10.3324/haematol.2020.257964 | Genome-wide CRISPR screen identifies regulators of MAPK and MTOR pathways mediating sorafenib resistance in acute myeloid leukemia |
| lestaurtinib | TYK2 | DOI: 10.1016/j.phrs.2016.07.038 | Janus kinase (JAK) inhibitors in the treatment of inflammatory and neoplastic diseases |
| cabozantinib | VEGFR1 | DOI: 10.2217/FON-2016-0358 | Cabozantinib in genitourinary malignancies |
| sunitinib | VEGFR1 | DOI: 10.2174/0929867324666171006165942 | Sunitinib in the Treatment of Thyroid Cancer |
| cabozantinib | VEGFR2 | DOI: 10.2217/FON-2016-0358 | Cabozantinib in genitourinary malignancies |
| Famitinib | VEGFR2 | PMID: 24238512 | Famitinib in metastatic renal cell carcinoma: a single center study |
| midostaurin | VEGFR2 | DOI: 10.1016/j.leukres.2011.05.006 | Tyrosine kinase inhibitors in the treatment of systemic mastocytosis |
| sorafenib | VEGFR2 | DOI: 10.1517/13543784.2012.665872 | Sorafenib in melanoma |
| Famitinib | VEGFR3 | PMID: 24238512 | Famitinib in metastatic renal cell carcinoma: a single center study |
| sorafenib | VEGFR3 | DOI: 10.1517/13543784.2012.665872 | Sorafenib in melanoma |
| sunitinib | VEGFR3 | DOI: 10.2174/0929867324666171006165942 | Sunitinib in the Treatment of Thyroid Cancer |
| sunitinib | VHL | DOI: 10.3390/cancers16010034 | Sunitinib Treatment of VHL C162F Cells Slows Down Proliferation and Healing Ability via Downregulation of ZHX2 and Confers a Mesenchymal Phenotype |
| Famitinib | KIT | DOI: 10.1158/1078-0432.CCR-21-4313 | Famitinib with Camrelizumab and Nab-Paclitaxel for Advanced Immunomodulatory Triple-Negative Breast Cancer (FUTURE-C-Plus): An Open-Label, Single-Arm, Phase II Trial |
| Famitinib | PDGFRA | DOI: 10.3109/09553002.2015.1062574 | Famitinib enhances nasopharyngeal cancer cell radiosensitivity by attenuating radiation-induced phosphorylation of platelet-derived growth factor receptor and c-kit and inhibiting microvessel formation |
| Famitinib | PDGFRB | DOI: 10.3892/ol.2016.4909 | Famitinib exerted powerful antitumor activity in human gastric cancer cells and xenografts |
| Famitinib | VEGFA | DOI: 10.1111/1759-7714.14689 | Famitinib enhances the antitumor effect of radioimmunotherapy in murine lung cancer |
| Famitinib | VEGFR3 | DOI: 10.1517/13543784.2016.1161754 | Novel anti-angiogenic therapeutic strategies in colorectal cancer |
| Famitinib | VEGFR2 | DOI: 10.1007/s00280-014-2505-x | Hypothyroidism as a potential biomarker of efficacy of famitinib, a novel VEGFR-2 inhibitor in metastatic breast cancer |
| Famitinib | CD8 | DOI: 10.1111/1759-7714.14689 | Famitinib enhances the antitumor effect of radioimmunotherapy in murine lung cancer |
| Famitinib | CYP3A4 | DOI: 10.1111/bph.12047 | Metabolism and bioactivation of famitinib, a novel inhibitor of receptor tyrosine kinase, in cancer patients |
| Famitinib | KAT6A | DOI: 10.1158/1078-0432.CCR-21-4313 | Famitinib with Camrelizumab and Nab-Paclitaxel for Advanced Immunomodulatory Triple-Negative Breast Cancer (FUTURE-C-Plus): An Open-Label, Single-Arm, Phase II Trial |
| Famitinib | VEGFC | DOI: 10.1517/13543784.2016.1161754 | Novel anti-angiogenic therapeutic strategies in colorectal cancer |
| Famitinib | VEGFR1 | DOI: 10.2147/CMAR.S215533 | Fruquintinib: a novel antivascular endothelial growth factor receptor tyrosine kinase inhibitor for the treatment of metastatic colorectal cancer |
| Famitinib | CYP3A5 | DOI: 10.1111/bph.12047 | Metabolism and bioactivation of famitinib, a novel inhibitor of receptor tyrosine kinase, in cancer patients |
| Famitinib | FLT3 | DOI: 10.1007/s00280-013-2282-y | Phase I study of the safety, pharmacokinetics and antitumor activity of famitinib |
| Famitinib | CD274 | DOI: 10.1186/s12943-022-01536-6 | Combined angiogenesis and PD-1 inhibition for immunomodulatory TNBC: concept exploration and biomarker analysis in the FUTURE-C-Plus trial |
| Famitinib | CYP1A1 | DOI: 10.1111/bph.12047 | Metabolism and bioactivation of famitinib, a novel inhibitor of receptor tyrosine kinase, in cancer patients |
| Famitinib | CYP1A2 | DOI: 10.1111/bph.12047 | Metabolism and bioactivation of famitinib, a novel inhibitor of receptor tyrosine kinase, in cancer patients |
| Famitinib | BRAF | DOI: 10.1002/cnr2.1770 | Neoadjuvant famitinib and camrelizumab, a new combined therapy allowing surgical resection of the primary site for anaplastic thyroid carcinoma |
| Famitinib | HIF1A | DOI: 10.1111/1759-7714.14689 | Famitinib enhances the antitumor effect of radioimmunotherapy in murine lung cancer |
| Famitinib | ERBB2 | DOI: 10.1007/s00280-014-2505-x | Hypothyroidism as a potential biomarker of efficacy of famitinib, a novel VEGFR-2 inhibitor in metastatic breast cancer |
| Famitinib | IGF1 | DOI: 10.1016/j.ando.2023.03.009 | Endocrine-related adverse conditions induced by tyrosine kinase inhibitors |
| Famitinib | PTH | DOI: 10.1016/j.ando.2023.03.009 | Endocrine-related adverse conditions induced by tyrosine kinase inhibitors |
| Famitinib | EGFR | DOI: 10.1111/1759-7714.13902 | Third-generation EGFR inhibitor HS-10296 in combination with famitinib, a multi-targeted tyrosine kinase inhibitor, exerts synergistic antitumor effects through enhanced inhibition of downstream signaling in EGFR-mutant non-small cell lung cancer cells |
| Famitinib | AKT1 | DOI: 10.1111/1759-7714.13902 | Third-generation EGFR inhibitor HS-10296 in combination with famitinib, a multi-targeted tyrosine kinase inhibitor, exerts synergistic antitumor effects through enhanced inhibition of downstream signaling in EGFR-mutant non-small cell lung cancer cells |
